# Supplementary material for: Proteomic risk score for early prediction of kidney disease progression in individuals with APOL1 high-risk genotypes
Source: Nat Med. 2026 Apr 15;32(5):1701–7. doi: 10.1038/s41591-026-04337-2 (PMC13190321; doi:10.1038/s41591-026-04337-2)
Supplement: Supplementary file 1 — Supplementary Figs. 1−3, Supplementary Tables 1 and 2, statistical analysis protocol and study protocol [file 41591_2026_4337_MOESM1_ESM.pdf]

nature medicine

Supplementary information https://doi.org/10.1038/s41591-026-04337-2

Proteomic risk score for early prediction of kidney disease progression in individuals with *APOL1* high-risk genotypes

In the format provided by the authors and unedited

Contents

Supplementary Figure 1. Correlation between kidney fibrosis and biomarker levels. 1

Supplementary Figure 2. Forest plots of hazard ratios and time-averaged AUC across subgroups in APOL1 high-risk individuals. 2

Supplementary Figure 3. Forest plots of hazard ratios and time-averaged AUC across subgroups in *APOL1* low-risk individuals. 3

Supplementary Table 1. Baseline characteristics of the training and testing cohort* 4

Supplementary Table 2. Comparison of Protein Hazard Ratios Between CRIC and PMBB Cohorts and Overlap 5

Statistical Analysis Protocol ....

Study Protocol ....

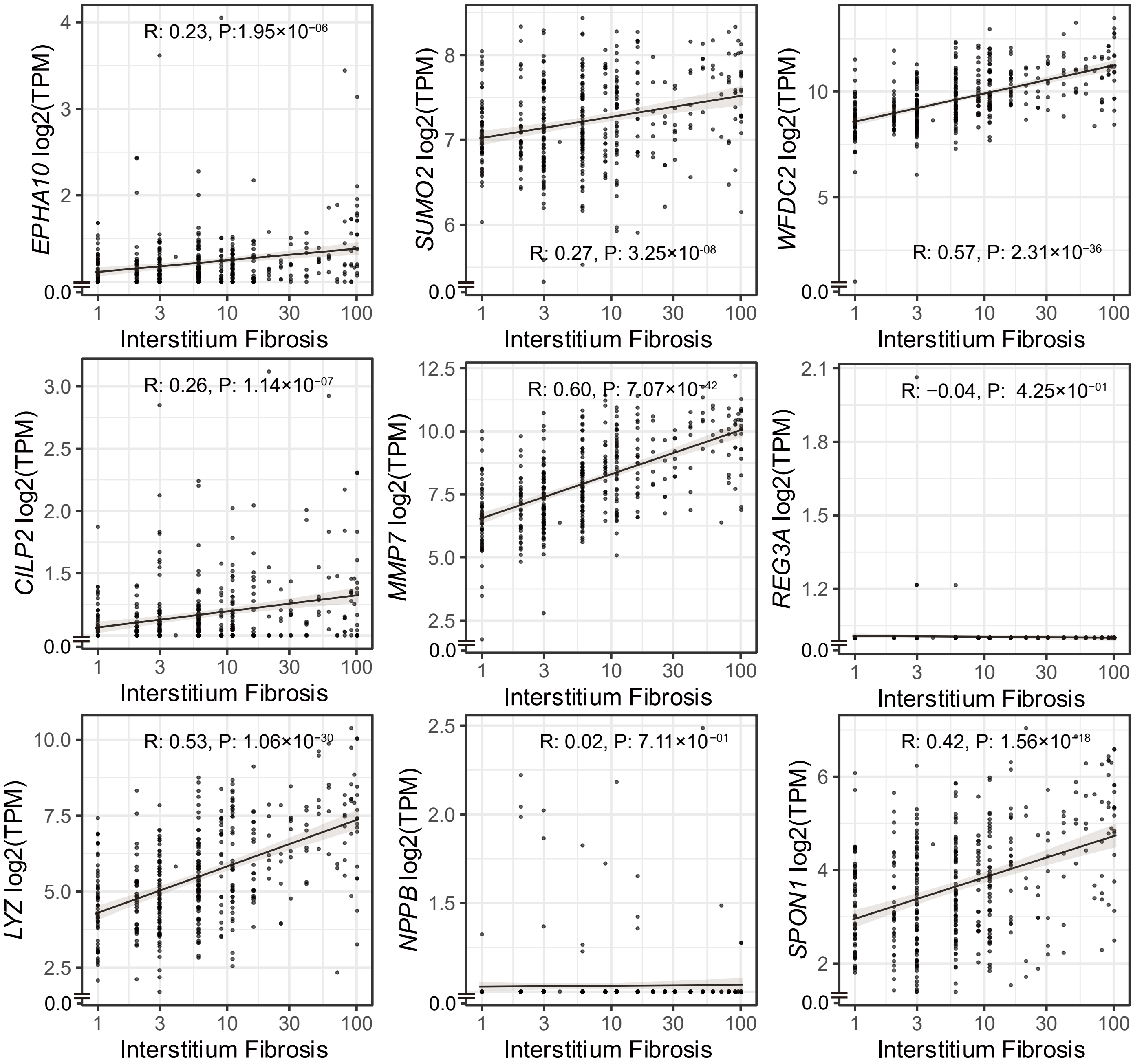

Supplementary Data Figure 1. Correlation between kidney fibrosis and biomarker levels.

Scatter plots showing the association between kidney interstitium fibrosis score (x-axis) and log_2-transformed gene expression levels (transcript per million, TPM. n= 474) of nine biomarkers (y-axis). Each panel displays the Pearson correlation coefficient (R) and corresponding two-sided P-value. Solid lines represent fitted values from linear regression models, and shaded areas indicate 95% confidence intervals.

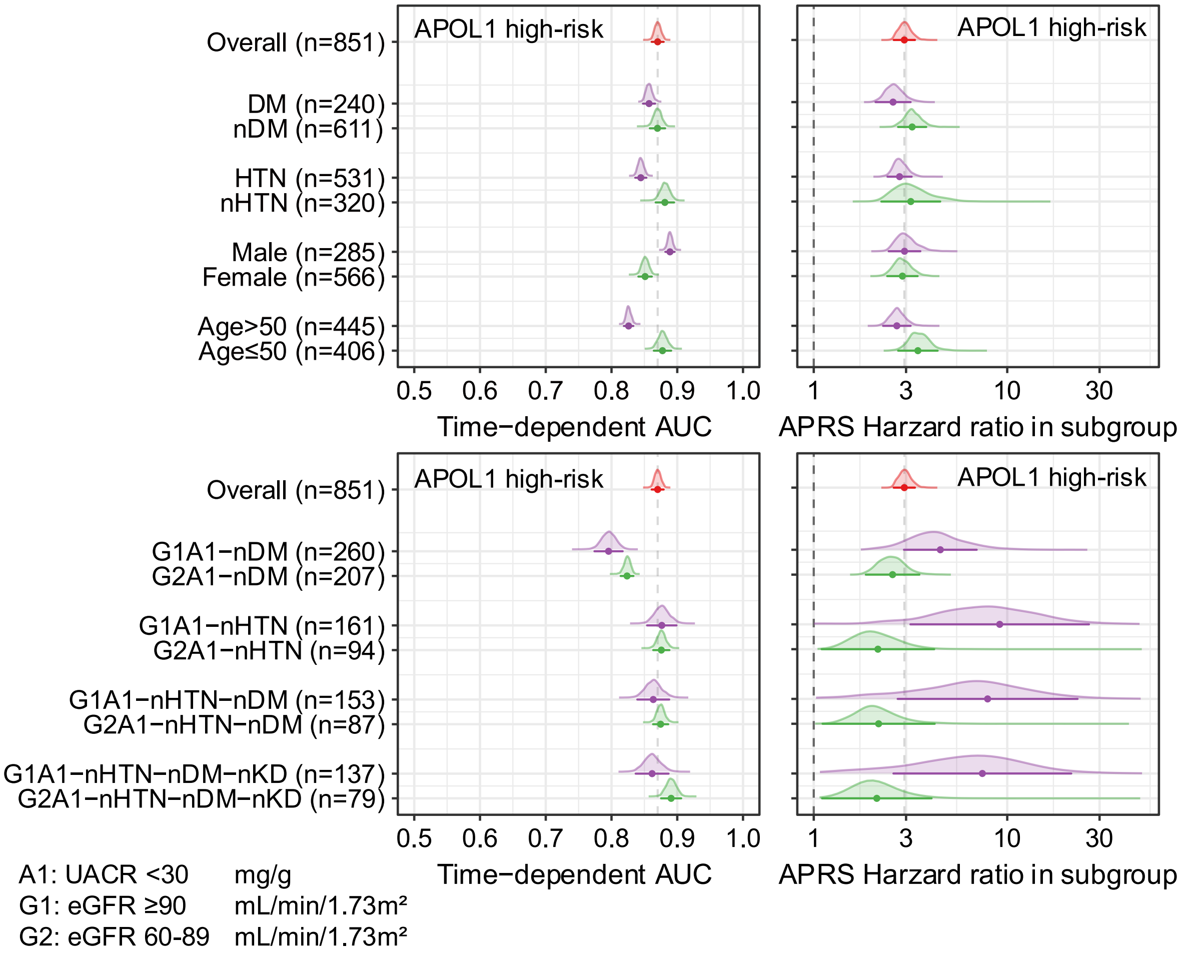

Supplementary Figure 2. Forest plots of hazard ratios and time-dependent AUC across subgroups in *APOL1* high-risk individuals.

(Right panel) Forest plot showing hazard ratios for the composite outcome in different subgroups. Points indicate hazard ratio estimates, horizontal lines represent 95% confidence intervals, and shaded density curves represent the distribution of hazard ratio estimates. The vertical dashed line corresponds to a hazard ratio of 1. (Left panel) Similar plots for the time-dependent AUC with 95% confidence intervals. DM, diabetes mellitus; HTN, hypertension. G1 and G2 denote eGFR categories (G1: eGFR ≥90; G2: 60–89 mL/min/1.73 m²). A1 denotes UACR categories (A1: <30 mg/g). Combined labels represent joint eGFR and UACR strata, and suffixes non-DM (nDM), non-HTN (nHTN); nKD excludes any history of kidney disease, including hematuria or ureteral disorders.

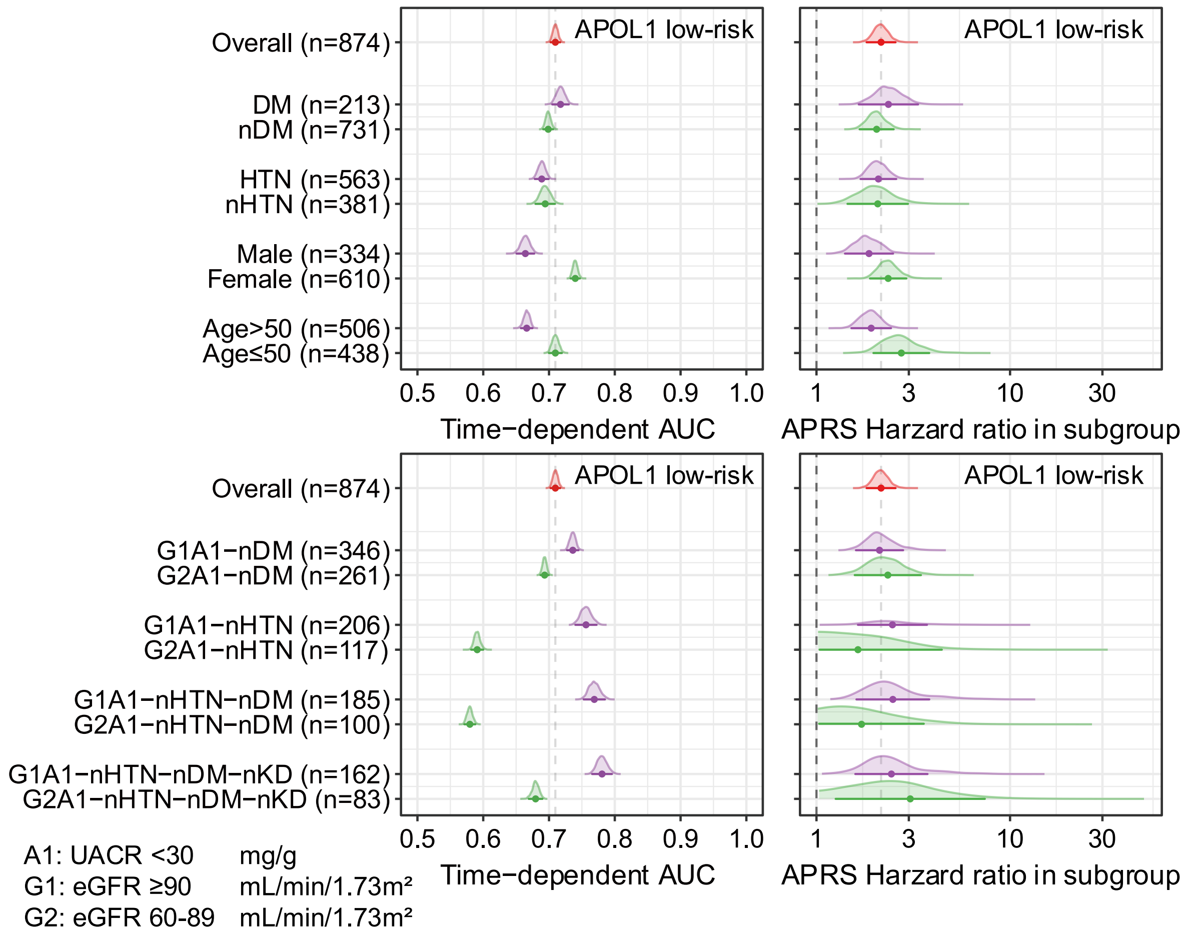

Supplementary Figure 3. Forest plots of hazard ratios and time-averaged AUC across subgroups in *APOL1* low-risk individuals.

(Right panel) Forest plot showing hazard ratios for the composite outcome in different subgroups. Points indicate hazard ratio estimates, horizontal lines represent 95% confidence intervals, and shaded density curves represent the distribution of hazard ratio estimates. The vertical dashed line corresponds to a hazard ratio of 1. (Left panel) Similar plots for the time-dependent AUC with 95% confidence intervals. DM, diabetes mellitus; HTN, hypertension. G1 and G2 denote eGFR categories (G1: eGFR ≥90; G2: 60–89 mL/min/1.73 m²). A1denotes UACR categories (A1: <30 mg/g). Combined labels represent joint eGFR and UACR strata, and suffixes non-DM (nDM), non-HTN (nHTN); nKD excludes any history of kidney disease, including hematuria or ureteral disorders.

Supplementary Table 1. Baseline characteristics of the training and testing cohort

	APOL1 high risk with eGFR ≥60 ml/min/1.73m²		
	Training	Testing	P
Number	680	171	
Age - yr	49.38±15.07	48.58±15.1	0.54
Female - no. (%)	452(66.47%)	114(66.67%)	
SBP - mmHg	128.61±17.73	128.27±18.09	0.82
DBP- mmHg	77.66±11.76	77.32±12.47	0.74
Body Mass Index - kg/m²	32.2±7.69	32.54±8.44	0.61
Hemoglobin A1c (%)	6.57±1.87	6.56±1.62	0.97
Creatinine (mg/dL)	0.88±0.21	0.87±0.22	0.92
Blood Urea Nitrogen (mg/dL)	12.99±4.87	13.25±5.44	0.55
eGFR - ml/min/1.73m²	90.73±16.94	90.03±17.47	0.63
UACR (IQR) - mg/g	17.23(11-31)	17.23(11-31)	0.67
30~299 - no. (%)	88(12.94)	24(14.03)	
≥ 300 - no. (%)	20(2.94)	5(2.92)	
Diagnostic group - no. (%)			
Hypertension	425(62.5)	106(61.99)	0.9
Diabetes Mellitus	186(27.3)	51(29.8)	0.52
Cardiovascular Disease	64(9.41)	14(8.19)	0.62
p.N264K	35(5.15)	5(2.92)	0.22
Event - no. (%)			
Composite Event	121(17.79)	32(18.71)	0.78
Deceased	48(7.06)	14(8.19)	0.61
Kidney event	96(14.12)	24(14.04)	0.98
≥ 40 % eGFR decline	89(13.09)	21(12.28)	0.78
Kidney transplantation	7(1.03)	3(1.75)	0.43
End-stage kidney disease	23(3.38)	3(1.75)	0.27
Dialysis	12(1.76)	3(1.75)	0.99
Follow-up time - yr	7.12±2.91	7.43±2.78	0.21
Time to event - yr	6.46±3.1	6.72±2.92	0.32

UACR: urine albumin-to-creatinine ratio; SBP: systolic blood pressure; DBP: diastolic blood pressure; eGFR: estimated glomerular filtration rate.

Supplementary Table 2. Comparison of Protein Hazard Ratios Between CRIC and PMBB Cohorts and Overlap

id	Target	Hazard Ratios per log2 (95%CI) adjusted for eGFR					Include in APRS
		CRIC (N. 65)	PMBB *APOL1* high-risk				
		eGFR<60	all	eGFR≥60	eGFR<60		
seq.11388.75	WFDC2	3.54 (2.98,4.22)	3.39(2.79-4.13)	3.73(2.89-4.81)	2.77(2.06-3.72)		Yes
seq.2789.26	MMP7	1.1 (1.06,1.14)	1.69(1.47-1.93)	1.86(1.51-2.30)	1.51(1.27-1.80)		Yes
seq.8841.65	CILP2	0.57 (0.5,0.64)	0.47(0.40-0.55)	0.41(0.33-0.52)	0.53(0.42-0.66)		Yes
seq.4297.62	SPON1		4.60(3.54-5.97)	4.87(3.43-6.92)	4.13(2.80-6.11)		Yes
seq.4920.10	LYZ		3.98(3.20-4.95)	4.28(3.22-5.68)	3.22(2.27-4.57)		Yes
seq.19555.1	SUMO2		2.85(2.42-3.36)	3.03(2.44-3.78)	2.43(1.90-3.11)		Yes
seq.6036.78	EPHA10		2.68(2.26-3.19)	2.85(2.26-3.61)	2.27(1.76-2.93)		Yes
seq.15304.1	REG3A		2.13(1.88-2.42)	2.36(1.99-2.81)	1.80(1.50-2.18)		Yes
seq.7655.11	NPPB		1.47(1.37-1.58)	1.68(1.52-1.86)	1.29(1.17-1.42)		Yes
seq.9234.8	TWSG1	4.95 (3.87,6.32)	9.22(6.31-13.46)	8.77(5.15-14.93)	8.46(4.74-15.1)		
seq.15453.3	AMBP	4.55 (3.61,5.74)	8.13(5.38-12.29)	7.87(4.29-14.43)	6.59(3.68-11.79)		
seq.6245.4	NECTIN2	4.46 (3.51,5.66)	3.63(2.41-5.46)	1.81(0.90-3.64)	4.35(2.44-7.74)		
seq.3438.10	FSTL3	4.26 (3.38,5.36)	3.89(3.10-4.86)	3.97(2.90-5.43)	3.42(2.43-4.81)		
seq.15472.16	LRP11	4.15 (3.32,5.18)	4.22(3.13-5.69)	4.21(2.70-6.55)	3.59(2.36-5.44)		
seq.8005.1	MXRA7	3.45 (2.93,4.07)	2.99(2.33-3.85)	3.11(2.10-4.61)	2.62(1.86-3.68)		
seq.5349.69	DLL1	3.37 (2.70,4.21)	3.64(2.73-4.85)	3.77(2.51-5.66)	3.16(2.09-4.78)		
seq.19575.4	EPOR	3.24 (2.77,3.77)	3.57(2.59-4.91)	3.68(2.28-5.94)	3.26(2.10-5.06)		
seq.19130.81	SERPINB8	3.19 (2.68,3.80)	2.71(2.17-3.39)	2.57(1.86-3.53)	2.62(1.89-3.62)		
seq.8587.21	SPINK14	3.06 (2.57,3.63)	2.53(1.82-3.51)	2.13(1.23-3.71)	2.34(1.53-3.58)		
seq.9468.8	LMAN2	2.92 (2.38,3.58)	5.81(4.39-7.69)	8.78(5.93-13.02)	3.76(2.57-5.49)		
seq.8480.29	EFEMP1	2.88 (2.37,3.49)	3.56(2.81-4.51)	4.81(3.49-6.63)	2.48(1.76-3.48)		
seq.5452.71	ASGR1	2.84 (2.41,3.36)	1.99(1.60-2.46)	1.66(1.15-2.42)	1.9(1.44-2.5)		
seq.15585.304	FBLN5	2.82 (2.30,3.46)	1.69(1.36-2.11)	1.90(1.41-2.57)	1.44(1.04-1.99)		
seq.14136.234	CD93	2.78 (2.24,3.45)	3.72(2.81-4.93)	3.83(2.56-5.73)	3.17(2.13-4.71)		
seq.3738.54	CSF1	2.66 (2.25,3.15)	1.75(1.28-2.39)	2.05(1.33-3.16)	1.51(0.96-2.38)		
seq.2997.8	JAM2	2.65 (2.15,3.28)	5.27(3.75-7.40)	6.32(3.61-11.05)	4.03(2.59-6.28)		
seq.9316.67	WFDC1	2.54 (2.14,3.02)	3.27(2.65-4.03)	3.75(2.76-5.08)	2.63(1.97-3.53)		
seq.2944.66	NBL1	2.52 (2.24,2.84)	3.62(2.81-4.67)	4.36(3.01-6.32)	2.97(2.11-4.19)		
seq.12488.9	MCTS1	2.49 (2.18,2.84)	1.90(1.61-2.24)	1.83(1.42-2.37)	1.77(1.43-2.2)		
seq.15640.54	TAGLN	2.37 (2.02,2.79)	2.84(2.43-3.31)	3.16(2.58-3.86)	2.36(1.86-2.98)		
seq.9021.1	HAVCR1	2.31 (2.15,2.48)	1.61(1.43-1.81)	1.93(1.63-2.30)	1.40(1.20-1.64)		
seq.12008.3	CD7	2.24 (1.96,2.56)	1.95(1.61-2.36)	1.61(1.19-2.16)	1.99(1.52-2.61)		
seq.2774.10	IL16	2.16 (1.83,2.56)	2.10(1.67-2.65)	1.60(1.15-2.23)	2.41(1.73-3.34)		
seq.14615.46	KRTAP2-4	1.91 (1.65,2.20)	1.78(1.38-2.30)	1.28(0.89-1.85)	2.09(1.46-2.98)		
seq.16322.10	MZB1	1.86 (1.67,2.08)	1.96(1.63-2.37)	2.35(1.76-3.13)	1.57(1.22-2.01)		
seq.2765.4	GDF11	1.85 (1.61,2.11)	0.90(0.70-1.15)	0.85(0.61-1.20)	1.01(0.71-1.45)		
seq.18841.1	SERPINB13	1.83 (1.62,2.06)	1.23(1.03-1.48)	1.05(0.80-1.36)	1.30(1.01-1.67)		
seq.7970.315	ART3	1.63 (1.45,1.84)	1.92(1.58-2.33)	1.63(1.19-2.22)	1.95(1.52-2.51)		
seq.4493.92	IL11	1.56 (1.24,1.97)	1.41(0.94-2.10)	1.84(1.05-3.22)	1.30(0.72-2.32)		
seq.5954.62	PTH	1.48 (1.36,1.61)	1.84(1.60-2.13)	1.90(1.52-2.38)	1.68(1.40-2.03)		
seq.6247.9	SIRPB1	1.43 (1.31,1.55)	1.92(1.63-2.28)	1.74(1.34-2.25)	1.86(1.49-2.34)		
seq.8219.14	ZG16	1.32 (1.21,1.43)	1.02(0.99-1.06)	1.01(0.96-1.07)	1.02(0.97-1.08)		
seq.5852.6	S100A12	1.29 (1.18,1.40)	1.44(1.24-1.68)	1.57(1.28-1.93)	1.42(1.14-1.77)		
seq.14175.78	SCP2D1	1.27 (1.19,1.35)	1.53(1.34-1.75)	1.73(1.40-2.14)	1.29(1.08-1.54)		
seq.7245.2	CELF2	1.21 (1.05,1.39)	1.12(0.98-1.28)	1.22(1.02-1.47)	1.09(0.90-1.32)		
seq.5090.49	LILRB1	0.91 (0.88,0.95)	1.00(0.9-1.12)	1.00(0.86-1.17)	1.00(0.86-1.16)		
seq.4914.10	CGA	0.83 (0.79,0.87)	0.91(0.85-0.97)	0.94(0.85-1.03)	0.88(0.80-0.96)		
seq.9322.15	RCN1	0.82 (0.75,0.89)	0.90(0.81-1.00)	0.91(0.79-1.05)	0.92(0.79-1.07)		
seq.11377.19	ADH7	0.81 (0.71,0.92)	0.70(0.55-0.89)	0.73(0.52-1.03)	0.70(0.49-0.98)		
seq.18179.56	SMS	0.8 (0.58,1.10)	0.38(0.21-0.69)	0.38(0.17-0.88)	0.32(0.13-0.78)		
seq.9185.15	TFF1	0.77 (0.71,0.82)	0.93(0.82-1.06)	0.98(0.82-1.19)	0.95(0.80-1.13)		
seq.9416.77	CPM	0.77 (0.68,0.87)	0.92(0.67-1.26)	0.88(0.57-1.34)	1.02(0.63-1.65)		

seq.12630.8	ARFIP2	0.75 (0.66,0.86)	1.08(0.83-1.41)	0.82(0.57-1.18)	1.50(1.01-2.23)	
seq.5632.6	CRTAC1	0.73 (0.66,0.81)	0.96(0.76-1.21)	0.99(0.70-1.39)	1.01(0.72-1.40)	
seq.5491.12	SPOCK2	0.72 (0.62,0.85)	0.62(0.50-0.77)	0.79(0.58-1.07)	0.55(0.40-0.77)	
seq.2961.1	PROC	0.68 (0.56,0.82)	0.26(0.18-0.37)	0.26(0.16-0.42)	0.28(0.16-0.50)	
seq.10521.10	MXRA8	0.67 (0.55,0.81)	1.92(1.39-2.65)	1.37(0.83-2.25)	2.23(1.47-3.38)	
seq.18274.2	SERF2	0.64 (0.49,0.83)	0.19(0.11-0.33)	0.11(0.05-0.27)	0.26(0.12-0.55)	
seq.14133.93	IL1R2	0.62 (0.53,0.73)	0.65(0.49-0.86)	0.74(0.50-1.09)	0.62(0.41-0.94)	
seq.10833.64	HHIP	0.61 (0.50,0.74)	1.00(0.73-1.35)	1.03(0.67-1.59)	1.08(0.70-1.65)	
seq.5091.28	LILRB2	0.60 (0.51,0.71)	1.37(0.95-1.96)	1.77(1.07-2.94)	1.15(0.69-1.92)	
seq.17495.141	SIRT3	0.59 (0.49,0.73)	0.54(0.43-0.69)	0.55(0.39-0.75)	0.53(0.37-0.77)	
seq.2706.69	SERPINA7	0.58 (0.45,0.76)	0.41(0.26-0.63)	0.44(0.23-0.81)	0.37(0.20-0.68)	
seq.9583.17	RNF24	0.53 (0.41,0.67)	0.65(0.43-0.99)	0.47(0.26-0.85)	0.79(0.44-1.43)	
seq.5107.7	NOTCH1	0.53 (0.37,0.74)	0.57(0.32-1.03)	0.40(0.17-0.92)	0.78(0.34-1.76)	
seq.19584.33	FGF9	0.50 (0.38,0.67)	0.26(0.17-0.40)	0.31(0.18-0.56)	0.26(0.13-0.51)	
seq.13554.78	REPIN1	0.49 (0.33,0.73)	0.33(0.18-0.58)	0.28(0.12-0.68)	0.36(0.16-0.78)	
seq.18896.23	HS6ST3	0.46 (0.36,0.60)	0.71(0.47-1.07)	0.59(0.33-1.06)	0.88(0.49-1.58)	
seq.5735.54	C1GALT1C1	0.45 (0.37,0.54)	0.18(0.10-0.34)	0.14(0.06-0.34)	0.30(0.12-0.75)	
seq.18380.78	ALB	0.40 (0.33,0.49)	2.04(1.26-3.30)	2.92(1.42-6.00)	1.41(0.74-2.69)	
seq.5359.65	PIM1	0.27 (0.20,0.37)	0.38(0.22-0.65)	0.51(0.25-1.04)	0.31(0.14-0.69)	

APOL1 Proteomic Risk Score (APRS) Study Protocol

Version / Date

Version: v3.0

Date: 2025-10-20

Corresponding Author

Name (Title): Katalin Susztak, MD, PhD

Affiliation: University of Pennsylvania

Email: ksusztak@pennmedicine.upenn.edu

Phone: (215)898-2009

Confidentiality Notice

This document contains proprietary protocol details and unpublished data. Distribution is limited to authorized reviewers and collaborators

Content

Summary of the change 1
1. Background 1
2. Objectives 1
3. Methods 2
3.1 Study Design and Setting 2
3.2 Penn Medicine BioBank (Primary Cohort) 2
3.3 ARIC Study (Validation Cohort) 2
3.4 UK Biobank (Validation Cohort) 3
3.5 Participant Eligibility 3
3.6 Study Endpoints and Follow-Up 3
3.7 Statistical Analysis 3
4. PMBB data source and PMBB-informed operations 4
4.1 Study independence and PMBB data source 4
4.2 Use of PMBB samples and EHR data 4
4.3 Informed consent (PMBB participants) 4
4.4 Withdrawal, limits, and participant choices 5
4.5 Privacy, security, and return of results 5
4.6 Governance and compliance 5
5. PMBB Biospecimen and Procedures 5
5.1 Subject Identification Numbers 5
5.2 Procedures 6
5.3 Biospecimen Workflow 6
6. Ethics and Oversight 6
7. Subject confidentiality and data security 7
7.1 Secure data storage 7
7.2 De-identification of data 7
7.3 Limited data access 7
7.4 Secure data transfer 7
7.5 Compliance with external database policies 8

Appendix 1 Propensity score matching for APOL1 high-risk and APOL1 low-risk with eGFR>60 cohort 9

Appendix 2 Propensity score matching for APOL1 high-risk and APOL1 low-risk with eGFR≤60 cohort 10

Appendix 3 Propensity score matching for all APOL1 high-risk and APOL1 low-risk cohort 11

Summary of the change

This revised protocol expands and refines our original design to improve risk prediction for APOL1-associated kidney disease. We now leverage multiple large cohorts (Penn Medicine BioBank, ARIC, UK Biobank) to increase sample size and diversity. Our focus is narrowed to individuals of African ancestry with APOL1 high-risk genotypes (two risk alleles) and preserved kidney function (eGFR ≥60 mL/min/1.73m^2 without significant albuminuria) at baseline, targeting the pre-chronic kidney disease phase.

1. Background

Chronic kidney disease (CKD) affects over 850 million people worldwide and over 800,000 in the U.S., imposing substantial morbidity, mortality, and health-care costs. Individuals of African ancestry suffer a disproportionately high ESKD burden, driven in part by APOL1 high-risk genotypes (two risk alleles, G1 and/or G2). An estimated 4–5 million Black Americans (and tens of millions globally) carry APOL1 high-risk genotypes, which confer ~5-fold higher risk of kidney failure. However, most APOL1–high-risk carriers remain healthy; only ≈20% progress to kidney failure. Emerging APOL1-targeted therapies (e.g., inaxaplin) offer promise for prevention, but their utility depends on identifying those high-risk carriers likely to progress before CKD develops. Currently, clinical risk equations (such as the Kidney Failure Risk Equation [KFRE]) perform poorly when eGFR is preserved. Broad plasma proteomic profiling can capture subclinical renal injury and dynamic biology that genomic or creatinine-based measures may miss. In our preliminary analyses, multiple circulating protein biomarkers were associated with long-term kidney outcomes in APOL1–high-risk individuals. We now propose to refine and validate APOL1 Proteomic Risk Score (APRS) for early risk stratification in this population.

2. Objectives

The primary and secondary objectives are to:

Develop and validate a plasma APOL1 proteomic risk score (APRS) for prediction of a composite kidney outcome (≥40% decline in eGFR, initiation of dialysis or kidney transplantation, or all-cause death) in APOL1 high-risk individuals with preserved kidney function.

Compare APRS performance (discrimination) to standard models including the KFRE, APOL1 genotype status, and CKD polygenic risk scores.

Externally validate APRS in independent, ancestry-matched cohorts (ARIC and UK Biobank) using harmonized outcomes.

Assess biological plausibility by evaluating the association of APRS protein components with kidney tissue pathology (e.g., interstitial fibrosis).

This material is the property of the University of Pennsylvania.

3. Methods

3.1 Study Design and Setting

This is a retrospective cohort study of adult participants with APOL1 high-risk genotypes drawn from the Penn Medicine BioBank (PMBB) and two external cohorts (ARIC, UK Biobank). The primary analysis will focus on APOL1–high-risk individuals of African ancestry with baseline eGFR>60 mL/min/1.73m^2 (preserved kidney function). The APRS will be derived in the PMBB cohort and validated in ARIC (African-American participants) and UK Biobank (African descent participants). All proteomic assays and outcome ascertainment will be harmonized across cohorts.

3.2 Penn Medicine BioBank (Primary Cohort)

PMBB is an EHR-linked biobank at the University of Pennsylvania with >40,000 participants, ~30% non-European ancestry. We will identify all African-American PMBB participants with APOL1 high-risk genotype (two risk alleles: G1/G1, G2/G2, or G1/G2), age ≥18, and baseline eGFR >60 mL/min/1.73m^2. APOL1 genotype was determined via whole-exome sequencing (Regeneron) or equivalent genotyping. Plasma proteomic profiles (7,549 proteins) were measured at baseline using the SomaScan aptamer platform. We have identified 1,113 eligible participants for the primary cohort (expanded from 550 in the prior dataset). Additional CKD participants with APOL1 high-risk genotype and 967 African ancestry APOL1 low-risk participants (267 newly added) and 698 European ancestry APOL1 low-risk were included as external reference. Baseline covariates include age, sex, eGFR (CKD-EPI 2021), and albumin-to-creatinine ratio (UACR). To better enable comparison between the African ancestry APOL1 high-risk and low-risk groups, propensity score matching was applied using age, sex, eGFR, UACR, diabetes, hypertension, and cardiovascular disease, with separate matching performed for participants with eGFR >60 and ≤60 mL/min/1.73 m^2 (Appendix 1 to 3).

3.3 ARIC Study (Validation Cohort)

The Atherosclerosis Risk in Communities (ARIC) Study is a prospective U.S. cohort of men and women (15,792 participants at baseline, 1987–1989). For validation, we will use African-American ARIC participants with APOL1 high-risk genotype. Specifically, 314 African-American APOL1 high-risk individuals who attended visit 2 (1990–1992) were included. An additional 2021 African-American individuals with APOL1 low-risk genotype and 8,602 European ancestry APOL1 low-risk participants were included as external reference. Genotyping (TaqMan assays) and SomaScan proteomics (5K proteins) were available on visit-2 samples. Follow-up (ascertainment of outcomes) is truncated at 10 years from

CONFIDENTIAL
This material is the property of the University of Pennsylvania.

visit 2 to harmonize with the PMBB analysis. All ARIC analyses were approved under study application MP4524.

3.4 UK Biobank (Validation Cohort)

The UK Biobank (UKB) is a large cohort of ~500,000 U.K. adults (ages 40–69 at enrollment, 2006–2010). We will validate APRS among UKB participants of African ancestry. Plasma proteomics were measured by the Olink platform in ~50,000 participants. Of these, 1,171 were of African descent, including 204 APOL1 high-risk individuals. Olink measurements will be log2-transformed and normalized; missing proteins (compared to SomaScan) will be imputed via multi-output penalized regression trained on PMBB data. Outcomes (eGFR, dialysis/transplant, mortality) are ascertained via linked clinical and registry data. Analyses are conducted under UKB Resource Application #273810.

3.5 Participant Eligibility

Inclusion Criteria: Adults (≥18 years) of genetically inferred African ancestry with APOL1 high-risk genotype (two risk alleles: G1/G1, G2/G2, or G1/G2); baseline eGFR >60 mL/min/1.73m^2; available baseline plasma proteomics; and at least one follow-up record (eGFR or relevant outcomes).

Exclusion Criteria: Prevalent end-stage kidney disease at baseline (on dialysis or prior kidney transplant); missing or ambiguous APOL1 genotype; failed proteomic assay; or insufficient key data for analysis.

3.6 Study Endpoints and Follow-Up

The primary endpoint is a composite defined as the earliest of: (1) ≥40% decline in eGFR from baseline; (2) onset of kidney failure (initiation of maintenance dialysis or kidney transplantation); or (3) all-cause mortality. These criteria mirror standard endpoints in CKD trials. Follow-up begins at the date of the index blood draw and continues until occurrence of the composite event, death, loss to follow-up, or end of study (up to ~10 years). Participants will be censored at the time of death if it precedes kidney endpoints, loss to follow-up, or at 10 years of follow-up to ensure comparability across cohorts.

3.7 Statistical Analysis

The APOL1–high-risk cohort will be randomly divided into an 80% training set and 20% test set (stratified by outcome incidence). In the training set, candidate proteins will be selected via an elastic-net penalized Cox proportional hazards regression (age, sex, baseline eGFR, and $\log_2$(UACR) included as unpenalized covariates). Tuning parameters (α, λ) will be chosen by 8-fold cross-validation. The final APRS model will include the nine selected proteins plus the four clinical covariates. Model discrimination will be quantified

This material is the property of the University of Pennsylvania.

by time-dependent AUC (tAUC) at prespecified time points and Harrell's C-index. Model performance will be compared to the KFRE (4-variable equation) and established CKD risk scores.

Proportional hazards assumptions for APRS and covariates will be tested using Schoenfeld residuals. Sensitivity analyses will address potential biases: we will apply inverse-probability-of-censoring weighting (IPCW) to account for informative loss-to-follow-up, computing weights within the training set. Feature stability will be assessed via bootstrap resampling. We will compute net benefit using decision-curve analysis (assuming equal weight for false positives and negatives). To gauge clinical utility, we will calculate the number needed to treat (NNT) to prevent one composite event under a hypothetical APOL1-targeted therapy: assuming a relative risk reduction of 27% (as reported for inaxaplin), NNT will be derived as the reciprocal of the absolute risk reduction. Comparisons of NNT between APRS-based risk stratification and standard models (KFRE) will be reported. All tests will be two-sided with α=0.05.

4. PMBB data source and PMBB-informed operations

4.1 Study independence and PMBB data source

The study relies entirely on biospecimens, and coded EHR data obtained from the PMBB (IRB #813913). All operational procedures and governance follow PMBB policies and practices. No new consent was obtained, as the study uses only data and samples already covered under PMBB consent.

4.2 Use of PMBB samples and EHR data

Cohort derivation relies on biospecimens, and coded clinical data provided by PMBB. Use of PMBB resources for this study follows PMBB's application and review workflow, requires local IRB approval, and is governed by executed Data Use Agreements (DUAs) and/or Material Transfer Agreements (MTAs) that specify minimum-necessary data elements and permitted uses. Any request to access re-identification keys or to perform linkages beyond the provided coded dataset requires additional PMBB approval and execution of a crosswalk/key-file agreement. Analyses of restricted datasets are conducted within approved secure computing environments as required by PMBB.

4.3 Informed consent (PMBB participants)

All participants whose samples and EHR data are included were originally enrolled in PMBB under PMBB's broad consent framework permitting future IRB-approved research on banked specimens and linked clinical data. If any additional, study-specific prospective enrollment is required, documented written informed consent will be obtained by trained study staff or via an IRB-approved electronic consenting

This material is the property of the University of Pennsylvania.

platform. Consent materials clearly describe study aims, intended uses of samples and data, foreseeable risks, voluntary participation, withdrawal options, and contact information.

4.4 Withdrawal, limits, and participant choices

Participants may withdraw at any time and will be offered tiered withdrawal options (for example: no further contact; unlinking of identifiers; request for destruction of remaining biospecimens where feasible). The consent will explicitly state that samples or data already released to approved investigators prior to a withdrawal decision generally cannot be recalled and may continue to be used under the terms of the original approvals. Operational handling of withdrawal requests will follow PMBB procedures for documenting and honoring the most recent consent decision.

4.5 Privacy, security, and return of results

Identifiable information will be stored separately from analytic datasets and maintained within institutional encrypted systems with role-based access controls, multifactor authentication, audit logging, and LIMS-linked specimen tracking. External sharing of data or specimens will occur only under DUAs/MTAs that explicitly prohibit attempts at re-identification; investigators must comply with monitoring and audit requirements and perform analyses within approved secure environments (e.g., Penn Medicine Academic Computing Services or equivalent). Individual research results will not be returned routinely; only analytically validated, clinically actionable findings that meet institutional criteria (confirmed in a CLIA-certified laboratory and accompanied by an IRB-approved return process and counseling when applicable) will be considered for disclosure.

4.6 Governance and compliance

All requests for sample or data release used by this protocol will be reviewed by the PMBB governance body (and/or the study governance committee) and require IRB-approved protocols and executed DUAs/MTAs. Investigators will provide periodic reports, comply with audits, and promptly report and remediate any data-security incidents in accordance with institutional policy. Cost-recovery fees for sample processing and shipping may apply per PMBB policy.

5. PMBB Biospecimen and Procedures

5.1 Subject Identification Numbers

This project utilizes samples and health data from participants previously enrolled in the PMBB. Because the PMBB consent explicitly allowed future use of these resources, no new consent process is required. When subjects joined the PMBB, they were given a unique study identifier. All related data and

This material is the property of the University of Pennsylvania.

biospecimens are labeled with this code rather than personal identifiers. Only approved members of the study team can access the secure linkage file that connects codes to individual identities.

5.2 Procedures

Within PMBB, patients from Penn Medicine and community volunteers provide medical history information and blood samples, including both those with kidney disease and healthy controls. Medical data are drawn from participant interviews and the electronic health record. Since participants originally agreed that their samples and data could be used for future studies, no direct re-contact is necessary. Study participants are identified on the basis of phenotype and genotype information already captured in PMBB. Their medical records are then reviewed to clarify disease characteristics, and relevant data are abstracted. All records retain only the minimal required Protected Health Information, which is stored on encrypted and password-protected devices. After abstraction and verification, direct identifiers are removed from analytic files, with a separate linkage file maintained under restricted access.

5.3 Biospecimen Workflow

PMBB biospecimens include blood, DNA, and protein. DNA is isolated and sequenced at certified core facilities, and analyses are performed by qualified bioinformatics staff. Sequencing is carried out using next-generation technologies, followed by computational alignment, filtering, and variant detection to identify potentially relevant rare mutations. Additional genotyping may be performed using standard array-based methods. Genotypes are compared against those of control participants. Plasma aliquots from PMBB are selected based on phenotypic and clinical characteristics and prepared by the PMBB Biospecimen Processing Core. Samples meeting quality and volume requirements are labeled with study-specific identifiers, shipped on dry ice in IATA-compliant packaging, and logged for traceability. Proteomic analyses are performed using high-throughput platforms such as SomaScan and resulting raw and processed data are returned to PMBB for secure storage and downstream analysis. The biospecimens and procedures involved in this project are covered under Penn Medicine Kidney Biobank protocol IRB#857403.

6. Ethics and Oversight

The data and biospecimen used in this study in PMBB were approved by the University of Pennsylvania Institutional Review Board (protocol 815796, 813913 and 857403) and all participants provided informed consent for genetic and EHR research. The validation approved by the University of Pennsylvania Institutional Review Board (protocol 855821). ARIC and UK Biobank investigations were conducted under their respective ethics approvals (ARIC field-center IRBs and UKB ethics; ARIC application MP4524; UKB

This material is the property of the University of Pennsylvania.

application 273810). All procedures will comply with the Declaration of Helsinki and applicable regulations. Participant privacy will be protected by using de-identified data, secure servers with encryption, and HIPAA-compliant protocols. Data use agreements restrict access to authorized personnel, and data transfer (for validation analyses) will employ secure, encrypted channels.

7. Subject confidentiality and data security

7.1 Secure data storage

All data obtained from external database will be stored on encrypted, password-protected Penn Medicine Academic Computing Services servers with access restricted to authorized personnel only. The servers are equipped with state-of-the-art firewalls and intrusion detection systems to safeguard against unauthorized access.

7.2 De-identification of data

The data available through many external databases has already undergone a strict de-identification process to remove personally identifiable information (PII) before being made available to approved researchers. However, in cases where the obtained data is not de-identified, we will implement our own de-identification process using industry-standard techniques such as data masking, pseudonymization, and tokenization. This process will be carried out by trained personnel and will be overseen by the project's data security officer to ensure compliance with Health Insurance Portability and Accountability Act (HIPAA) and other relevant regulations.

7.3 Limited data access

Access to the de-identified data will be granted on a need-to-know basis, with only the principal investigator and key research personnel having direct access. All individuals with data access will be required to undergo training in data security and confidentiality protocols and will sign confidentiality agreements.

7.4 Secure data transfer

When data sharing is necessary for collaboration or validation purposes, we will employ secure file transfer protocols from Upenn (https://www.isc.upenn.edu/security/secure-share) and encrypt all data in transit. Data will only be shared with collaborators who have obtained the necessary institutional review board (IRB) approvals and have signed data use agreements outlining the terms and conditions of data access and usage.

CONFIDENTIAL
This material is the property of the University of Pennsylvania.

7.5 Compliance with external database policies

We will strictly adhere to all data use and confidentiality policies set forth by external databases, including the Genomic Data Sharing (GDS) Policy and the NIH Security Best Practices for Controlled-Access Data. In cases where the policies of the external database do not fully address the handling of non-de-identified data, we will develop and implement our own policies that prioritize the protection of subject privacy and confidentiality. These policies will be reviewed and updated regularly to ensure they remain in line with the current best practices and regulatory requirements. Regular audits will be conducted to ensure ongoing compliance with both external and internal data security and confidentiality policies.

Appendix 1 Propensity score matching for APOL1 high-risk and APOL1 low-risk with eGFR>60 cohort

	APOL1 high-risk	APOL1 low-risk			
		Initial cohort		Propensity score matching	
Number	851	944	P value	874	P value
Age - yr	49.22±15.07	50.37±13.81	0.09	49.9±14.03	0.33
Female - no. (%)	566(66.51%)	610(64.62%)	0.4	568(64.99%)	0.51
SBP - mmHg	128.54±17.79	129.34±17.46	0.34	129.3±17.41	0.37
DBP- mmHg	77.59±11.9	77.51±11.44	0.88	77.42±11.47	0.76
Body Mass Index - kg/m²	32.27±7.84	32.06±7.82	0.57	32.29±7.83	0.95
Hemoglobin A1c (%)	6.57±1.81	6.36±1.36	0.11	6.39±1.39	0.19
Creatinine (mg/dL)	0.88±0.21	0.84±0.19	<0.01	0.86±0.18	0.07
Blood Urea Nitrogen (mg/dL)	13.04±4.98	12.44±4.34	<0.01	12.56±4.39	0.03
eGFR - ml/min/1.73m²	90.59±17.04	93.04±16.38	<0.01	91.96±16.39	0.09
UACR (IQR) - mg/g	17.23(11-31)	15.31(12-24)	0.02	15.31(12-24)	0.05
30~299 - no. (%)	112(13.16)	153(16.21)		138(15.79)	
≥ 300 - no. (%)	25(2.93)	24(2.54)		23(2.63)	
Red Blood Cells (×10⁶/µL)	4.32±0.63	4.35±0.61	0.43	4.34±0.61	0.44
White Blood Cells (×10³/µL)	7.36±3.46	7.21±3.08	0.32	7.21±3.12	0.33
Hemoglobin (g/dL)	12.31±1.78	12.51±1.81	0.02	12.51±1.81	0.02
Neutrophils (×10³/µL)	4.55±2.61	4.4±2.33	0.2	4.43±2.35	0.3
Monocytes (×10³/µL)	0.56±0.25	0.54±0.22	0.2	0.54±0.22	0.22
Lymphocytes (×10³/µL)	1.92±0.82	1.91±0.82	0.85	1.9±0.81	0.67
Eosinophils (×10³/µL)	0.15±0.13	0.14±0.12	0.21	0.14±0.12	0.15
Platelets (×10³/µL)	255.28±85.21	251.08±96.84	0.33	249.55±97.05	0.19
Chloride (mmol/L)	103.73±3.29	103.72±3.21	0.9	103.69±3.19	0.75
Magnesium (mg/dL)	1.94±0.22	1.93±0.24	0.97	1.94±0.24	0.99
Potassium (mmol/L)	4.13±0.39	4.11±0.37	0.17	4.11±0.37	0.21
Calcium (mg/dL)	9.25±0.55	9.28±0.57	0.23	9.29±0.56	0.09
Sodium (mmol/L)	138.42±2.56	138.63±2.54	0.08	138.61±2.54	0.11
Glucose (mg/dL)	111.91±50.47	106.9±45.11	0.03	107.82±46.28	0.08
Alkaline Phosphatase (U/L)	76.37±36.01	75.69±32.41	0.67	75.32±32.46	0.52
Alanine Aminotransferase (U/L)	23.08±35.55	22.53±23.19	0.7	22.88±23.9	0.89
Aspartate Aminotransferase (U/L)	26.87±65.87	24.6±30.24	0.34	24.86±31.18	0.41
Low-Density Lipoprotein Cholesterol (mg/dL)	105.32±33.2	102.87±33.5	0.4	103.16±33.81	0.47
High-Density Lipoprotein Cholesterol (mg/dL)	48.72±15.45	50.86±14.31	0.09	51.04±14.18	0.07
Total Cholesterol (mg/dL)	180.1±51.87	177.98±45.39	0.61	178.53±45.77	0.71
Total Bilirubin (mg/dL)	0.59±0.41	0.58±0.36	0.51	0.58±0.36	0.56
Prothrombin Time (sec)	14.44±4.43	14.04±3.72	0.22	14.04±3.79	0.23
Activated Partial Thromboplastin Time (sec)	30.25±7.0	29.91±7.01	0.56	30.04±7.22	0.73
Albumin (g/dL)	3.98±0.47	4.01±0.41	0.14	4.01±0.41	0.13
Thyroid-Stimulating Hormone (µIU/mL)	2.22±5.26	1.78±1.98	0.22	1.77±1.98	0.23
Triglycerides (mg/dL)	119.47±180.06	113.44±139.57	0.65	113.31±143.06	0.66
Diagnostic group - no. (%)					
Hypertension	531(62.4%)	563(59.64%)	0.23	532(60.87%)	0.51
Diabetes Mellitus	237(27.85%)	211(22.35%)	<0.01	211(24.14%)	0.08
Cardiovascular Disease	78(9.17%)	109(11.55%)	0.1	100(11.44%)	0.12
p.N264K	40(4.7%)	60(6.36%)	0.09	56(6.41%)	0.09
Event - no. (%)					
Composite Event	153(17.98%)	142(15.04%)	0.09	135(15.45%)	0.16
Deceased	62(7.29%)	73(7.73%)	0.72	68(7.78%)	0.7
Kidney event	120(14.1%)	90(9.53%)	<0.01	86(9.84%)	<0.01
Follow-up time - yr	7.18±2.89	7.62±2.69	<0.01	7.64±2.68	<0.01
Time to event - yr	6.51±3.07	7.22±2.86	<0.01	7.24±2.86	<0.01

Appendix 2 Propensity score matching for APOL1 high-risk and APOL1 low-risk with eGFR≤60 cohort

	APOL1 high-risk	APOL1 low-risk			
		Initial cohort		Propensity score matching	
Number	262	51	P value	38	P value
Age - yr	57.73±15.84	54.68±14.61	0.2	55.41±15.38	0.4
Female - no. (%)	135(51.53%)	36(70.59%)	0.01	25(65.79%)	0.1
SBP - mmHg	130.87±19.81	131.84±17.66	0.74	133.55±18.5	0.43
DBP- mmHg	75.23±12.26	79.04±12.18	0.04	78.75±13.7	0.08
Body Mass Index - kg/m²	30.93±7.58	32.0±8.56	0.37	32.71±8.39	0.18
Hemoglobin A1c (%)	6.66±1.81	6.03±0.88	0.19	6.37±0.86	0.63
Creatinine (mg/dL)	1.99±0.95	1.56±0.79	<0.01	1.68±0.87	0.06
Blood Urea Nitrogen (mg/dL)	28.69±14.44	25.27±13.93	0.12	27.63±15.33	0.67
eGFR - ml/min/1.73m²	38.19±16.67	45.99±14.5	<0.01	43.46±15.97	0.07
UACR (IQR) - mg/g	31.67(15-124)	17.23(12-45)	0.14	24.12(12-62)	0.26
30~299 - no. (%)	51(20.23)	12(23.53)		11(28.95)	
≥ 300 - no. (%)	35(13.89)	5(9.80)		5(13.16)	
Red Blood Cells (×10⁶/µL)	3.98±0.7	4.08±0.67	0.37	4.01±0.7	0.79
White Blood Cells (×10³/µL)	7.44±3.54	7.56±2.87	0.83	7.73±2.87	0.63
Hemoglobin (g/dL)	11.67±1.91	11.87±1.89	0.5	11.54±1.85	0.7
Neutrophils (×10³/µL)	4.9±2.55	4.93±2.82	0.93	5.05±2.92	0.73
Monocytes (×10³/µL)	0.61±0.27	0.58±0.28	0.48	0.55±0.27	0.24
Lymphocytes (×10³/µL)	1.67±0.78	1.61±0.81	0.67	1.57±0.64	0.46
Eosinophils (×10³/µL)	0.17±0.15	0.17±0.12	0.81	0.17±0.12	0.99
Platelets (×10³/µL)	231.46±74.53	217.75±69.81	0.23	225.18±63.47	0.62
Chloride (mmol/L)	103.42±4.48	103.73±4.88	0.66	103.37±4.84	0.95
Magnesium (mg/dL)	2.02±0.25	2.01±0.37	0.95	2.07±0.44	0.6
Potassium (mmol/L)	4.29±0.57	4.42±0.43	0.15	4.42±0.46	0.21
Calcium (mg/dL)	9.25±0.61	9.2±0.68	0.58	9.11±0.73	0.21
Sodium (mmol/L)	138.64±3.06	138.41±3.01	0.63	138.18±3.2	0.4
Glucose (mg/dL)	119.12±58.54	122.76±56.47	0.68	132.87±60.75	0.18
Alkaline Phosphatase (U/L)	85.99±38.93	92.55±64.64	0.33	99.18±72.6	0.09
Alanine Aminotransferase (U/L)	22.54±28.12	26.63±26.49	0.34	29.39±30.08	0.17
Aspartate Aminotransferase (U/L)	25.71±23.7	27.76±27.62	0.58	29.68±31.57	0.36
Low-Density Lipoprotein Cholesterol (mg/dL)	96.96±36.72	121.36±24.6	0.04	118.86±28.92	0.13
High-Density Lipoprotein Cholesterol (mg/dL)	51.73±20.71	52.17±13.15	0.94	46.38±11.65	0.48
Total Cholesterol (mg/dL)	179.19±79.81	189.08±42.36	0.68	180.88±50.22	0.95
Total Bilirubin (mg/dL)	0.66±0.46	0.75±0.63	0.22	0.74±0.59	0.33
Prothrombin Time (sec)	15.78±7.56	14.72±5.36	0.55	14.73±6.15	0.62
Activated Partial Thromboplastin Time (sec)	32.87±13.72	29.35±3.87	0.33	29.42±3.98	0.39
Albumin (g/dL)	3.84±0.54	3.83±0.64	0.91	3.81±0.68	0.75
Thyroid-Stimulating Hormone (µIU/mL)	3.37±9.87	1.65±1.01	0.57	1.88±1.07	0.69
Triglycerides (mg/dL)	108.85±47.9	114.57±60.53	0.7	128.89±71.13	0.27
Diagnostic group - no. (%)					
Hypertension	223(85.11%)	29(56.86%)	<0.01	28(73.68%)	0.08
Diabetes Mellitus	98(37.4%)	13(25.49%)	0.1	12(31.58%)	0.49
Cardiovascular Disease	38(14.5%)	5(9.8%)	0.37	5(13.16%)	0.83
p.N264K	5(1.91%)	2(3.92%)	0.38	2(5.26%)	0.2
Event - no. (%)					
Composite Event	145(55.34%)	6(11.76%)	<0.01	3(7.89%)	<0.01
Deceased	57(21.76%)	4(7.84%)	0.02	2(5.26%)	0.02
Kidney event	125(47.71%)	4(7.84%)	<0.01	2(5.26%)	<0.01
Follow-up time - yr	6.67±3.5	7.19±2.8	0.31	7.42±2.77	0.2
Time to event - yr	4.02±3.57	6.85±3.09	<0.01	7.25±2.82	<0.01

This material is the property of the University of Pennsylvania.

Appendix 3 Propensity score matching for all APOL1 high-risk and APOL1 low-risk cohort

	APOL1 high-risk	APOL1 low-risk			
		Initial cohort		Propensity score matching	
Number	1113	995	P value	912	P value
Age - yr	51.22±15.67	50.59±13.88	0.33	50.13±14.12	0.1
Female - no. (%)	701(62.98%)	646(64.92%)	0.35	593(65.02%)	0.34
SBP - mmHg	129.09±18.3	129.47±17.47	0.63	129.48±17.47	0.63
DBP- mmHg	77.04±12.02	77.59±11.47	0.28	77.56±11.54	0.32
Body Mass Index - kg/m²	31.95±7.8	32.05±7.86	0.77	32.31±7.85	0.31
Hemoglobin A1c (%)	6.59±1.81	6.35±1.35	0.04	6.39±1.38	0.11
Creatinine (mg/dL)	1.14±0.68	0.88±0.3	<0.01	0.89±0.3	<0.01
Blood Urea Nitrogen (mg/dL)	16.73±10.59	13.1±5.97	<0.01	13.19±6.09	<0.01
eGFR - ml/min/1.73m²	78.26±27.96	90.63±19.31	<0.01	89.94±19.02	<0.01
UACR (IQR) - mg/g	17.23(11-44)	15.31(12-24)	<0.01	15.31(12-24)	<0.01
30~299 - no. (%)	163(14.64)	165(16.58)		149(16.34)	
≥ 300 - no. (%)	60(5.39)	29(2.91)		28(3.07)	
Red Blood Cells (×10⁶/μL)	4.24±0.66	4.33±0.62	<0.01	4.33±0.62	<0.01
White Blood Cells (×10³/μL)	7.38±3.48	7.23±3.07	0.28	7.23±3.11	0.3
Hemoglobin (g/dL)	12.16±1.83	12.48±1.82	<0.01	12.47±1.82	<0.01
Neutrophils (×10³/μL)	4.63±2.6	4.43±2.36	0.06	4.45±2.38	0.11
Monocytes (×10³/μL)	0.57±0.26	0.55±0.23	0.02	0.54±0.23	0.02
Lymphocytes (×10³/μL)	1.86±0.82	1.9±0.82	0.3	1.89±0.8	0.42
Eosinophils (×10³/μL)	0.16±0.13	0.15±0.12	0.05	0.14±0.12	0.03
Platelets (×10³/μL)	249.67±83.4	249.37±95.89	0.94	248.54±95.98	0.78
Chloride (mmol/L)	103.66±3.6	103.72±3.31	0.71	103.67±3.27	0.94
Magnesium (mg/dL)	1.96±0.23	1.94±0.24	0.34	1.94±0.25	0.41
Potassium (mmol/L)	4.17±0.44	4.12±0.38	<0.01	4.12±0.38	<0.01
Calcium (mg/dL)	9.25±0.57	9.27±0.57	0.28	9.28±0.57	0.15
Sodium (mmol/L)	138.47±2.69	138.62±2.57	0.19	138.59±2.57	0.29
Glucose (mg/dL)	113.61±52.54	107.71±45.86	<0.01	108.87±47.2	0.03
Alkaline Phosphatase (U/L)	78.64±36.93	76.55±34.94	0.18	76.32±35.31	0.15
Alanine Aminotransferase (U/L)	22.95±33.94	22.74±23.38	0.87	23.15±24.2	0.88
Aspartate Aminotransferase (U/L)	26.6±58.73	24.76±30.11	0.37	25.06±31.19	0.48
Low-Density Lipoprotein Cholesterol (mg/dL)	103.35±34.18	103.52±33.38	0.95	103.54±33.74	0.95
High-Density Lipoprotein Cholesterol (mg/dL)	49.41±16.82	50.91±14.26	0.22	50.92±14.12	0.23
Total Cholesterol (mg/dL)	179.89±59.36	178.39±45.26	0.72	178.6±45.8	0.76
Total Bilirubin (mg/dL)	0.61±0.42	0.59±0.38	0.25	0.59±0.38	0.24
Prothrombin Time (sec)	14.82±5.52	14.08±3.82	0.03	14.07±3.9	0.04
Activated Partial Thromboplastin Time (sec)	30.97±9.38	29.89±6.89	0.09	30.01±7.11	0.15
Albumin (g/dL)	3.95±0.49	4.0±0.43	<0.01	4.0±0.43	<0.01
Thyroid-Stimulating Hormone (μIU/mL)	2.55±6.9	1.77±1.95	0.08	1.78±1.96	0.09
Triglycerides (mg/dL)	117.01±159.45	113.49±137.11	0.76	113.77±141.45	0.79
Diagnostic group - no. (%)					
Hypertension	754(67.74%)	592(59.5%)	<0.01	560(61.4%)	<0.01
Diabetes Mellitus	335(30.1%)	224(22.51%)	<0.01	223(24.45%)	<0.01
Cardiovascular Disease	116(10.42%)	114(11.46%)	0.45	105(11.51%)	0.43
p.N264K	45(4.04%)	62(6.23%)	0.01	58(6.36%)	0.01
Event - no. (%)					
Composite Event	298(26.77%)	148(14.87%)	<0.01	138(15.13%)	<0.01
Deceased	119(10.69%)	77(7.74%)	0.02	70(7.68%)	0.02
Kidney event	245(22.01%)	94(9.45%)	<0.01	88(9.65%)	<0.01
Follow-up time - yr	7.06±3.05	7.59±2.7	<0.01	7.64±2.69	<0.01
Time to event - yr	5.93±3.36	7.2±2.87	<0.01	7.24±2.85	<0.01

This material is the property of the University of Pennsylvania.

Statistical Analysis Protocol

Version / Date

Version: v2.0

Date: 2025-08-22

Corresponding Author

Name (Title): Katalin Susztak, MD, PhD

Affiliation: University of Pennsylvania

Email: ksusztak@pennmedicine.upenn.edu

Phone: (215)898-2009

Confidentiality Notice

This document contains proprietary protocol details and unpublished data. Distribution is limited to authorized reviewers and collaborators.

Content

1 Primary aim 1
2 Background and Rationale 1
3 Cohorts, inclusion criteria, and analytic sets 1
4 Data elements, preprocessing, and QC 2
4.1Clinical variables 2
4.2 Proteomics (SOMAscan in PMBB) 2
5 Harmonization of Olink and SOMAscan 3
5.1 Standardization of Shared Proteins 3
5.2 Imputation of non-overlapping proteins 4
5.3 Robustness checks 4
6 Statistical methods 5
6.1 Missing Data Handling 5
6.2 Statistical Analysis for Multiplicity Correction 5
6.3 Cox proportional hazards model 6
6.4 Feature Selection and modeling 6
6.5 Number needed to treat (NNT) 7
7 Model Performance Evaluation 9
8 Sample Size and Power 13

1 Primary aim

To develop and externally validate a proteomic-clinical risk score (APRS) that predicts incident, clinically meaningful kidney outcomes among APOL1 high-risk (APOL1-HR) adults with preserved baseline kidney function, thereby enabling earlier identification and targeting for upstream intervention.

2 Background and Rationale

Chronic kidney disease remains a major global health burden, affecting an estimated 10–15% of the population worldwide. Individuals of African ancestry experience a disproportionately higher incidence of chronic kidney disease and progression to end-stage kidney disease. A key driver of this disparity is the presence of APOL1 high-risk alleles (G1 and G2)[1]. Individuals who carry two risk alleles (APOL1-HR genotype) face a significantly elevated risk of chronic kidney disease progression—yet most do not develop kidney disease, highlighting substantial phenotypic heterogeneity.

Although the APOL1 genotype is a well-established driver of kidney disease risk, not all carriers of HR alleles progress to end-stage kidney disease. Large-scale studies have estimated that ~20% of individuals with APOL1-HR may eventually develop kidney failure[2]. Current clinical tools, including the Kidney Failure Risk Equation[3] and other creatinine-based or albuminuria-based formulas, often underperform in predicting kidney outcomes at earlier stages (estimated glomerular filtration rate, eGFR ≥60 mL/min/1.73m²). Consequently, there is an urgent need for noninvasive biomarkers that can distinguish individuals at greatest risk, enabling timely interventions[4] (e.g., APOL1 inhibitors, stricter blood pressure control, etc.).

High-throughput proteomics (e.g., SomaScan®, Olink®) has become increasingly accessible for large patient cohorts[5]. Circulating protein levels may reflect underlying pathophysiological mechanisms more directly than genetic risk alone. Our preliminary data in African American patients with APOL1-HR genotypes identified multiple protein signals that correlate with adverse renal outcomes. In this study, we comprehensively assess the utility of these protein markers in a combined protein-clinical model, test their incremental value beyond genetic or clinical risk factors, and validate the model in independent cohorts.

3 Cohorts, inclusion criteria, and analytic sets

Primary derivation set (APOL1-HR, preserved eGFR):

> Inclusion: APOL1 genotype = high-risk (two risk alleles: G1/G1, G2/G2, G1/G2), available plasma proteomics (SomaScan platform), baseline eGFR > 60 mL/min/1.73 m² measured at or 2 months before or 1 month after of sampling, available UACR (or imputed as specified).

Final sample size in derivation set: N = 851. Baseline descriptive statistics, event counts, and follow-up summaries are provided in manuscript. Key cohort summary (derivation APOL1-HR preserved-eGFR): mean age 49.2 years; female proportion 66.5%; mean eGFR 90.6 mL/min/1.73 m²; median UACR 17.23 mg/g; composite event rate 17.98% (n 153); mean time to event 6.51 years; median follow-up 7.3 years.

Secondary analytic sets:

APOL1-HR with existing CKD at baseline: 262 participants included for comparison and benchmarking of APRS behavior in established disease.

APOL1-low-risk extension: an additional 995 participants to evaluate specificity and cross-genotype performance.

EUR comparison set (review-only): N = 514 (used for method comparisons but not central to the APOL1-focused primary aims).

External validation cohorts

ARIC (Atherosclerosis Risk in Communities): African-ancestry subset with SomaScan proteomics (where available).

UK Biobank (UKB): African-ancestry subset with Olink proteomics (smaller targeted panels). Because platforms differ, apply the prespecified cross-platform mapping and multi-output imputation.

4 Data elements, preprocessing, and QC

4.1Clinical variables

- Demographics: age (years), sex, genetic ancestry.
- Baseline labs: serum creatinine (to compute eGFR using CKD-EPI 2021), UACR (spot urine albumin/creatinine; log-transform for modeling), SBP/DBP, BMI, etc. For continuous labs, use the value closest to proteomic draw 2 months before or 1 month after sampling. For multiple measurements in the window, use the value nearest the draw date. Convert UACR to mg/g and log2 or natural log as specified (log2-UACR used in final model).
- Comorbidities: diabetes mellitus status, hypertension, cardiovascular disease history, etc.

4.2 Proteomics (SOMAscan in PMBB)

The proteomic assays for this study were performed in two runs (batches) using identical laboratory protocols, instrumentation, reagent lots where possible, and operator procedures. Because the data were produced in two batches, we evaluated potential batch effects before modeling. The initial showed apparent separation by assay

run. Investigation revealed that samples in Run 2 had systematically higher baseline eGFR, so run was confounded with this clinical variable.

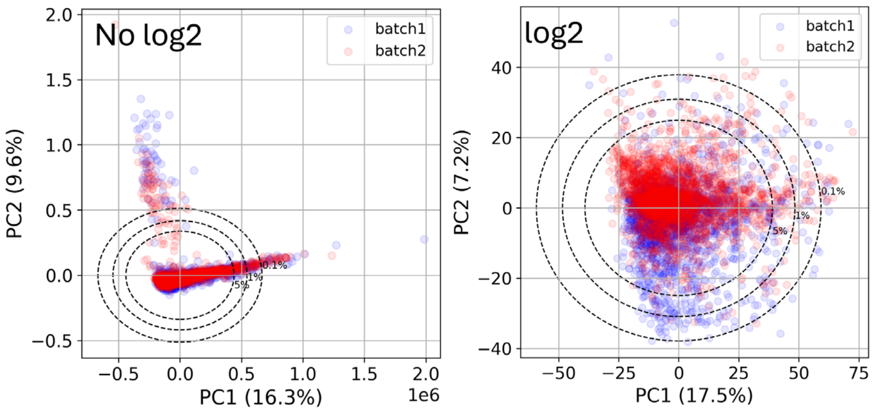

To determine whether the observed separation reflected technical batch bias or underlying biological differences, we adjusted each protein for baseline. PCA on the eGFR-adjusted residual no longer showed run-driven clustering, and formal tests of association between PC1 and PC2 and run were non-significant after adjustment. Based on these diagnostics, we concluded that the initial run separation was driven by biological differences in eGFR rather than a technical batch effect; accordingly, no additional batch-correction was applied in the derivation analyses.

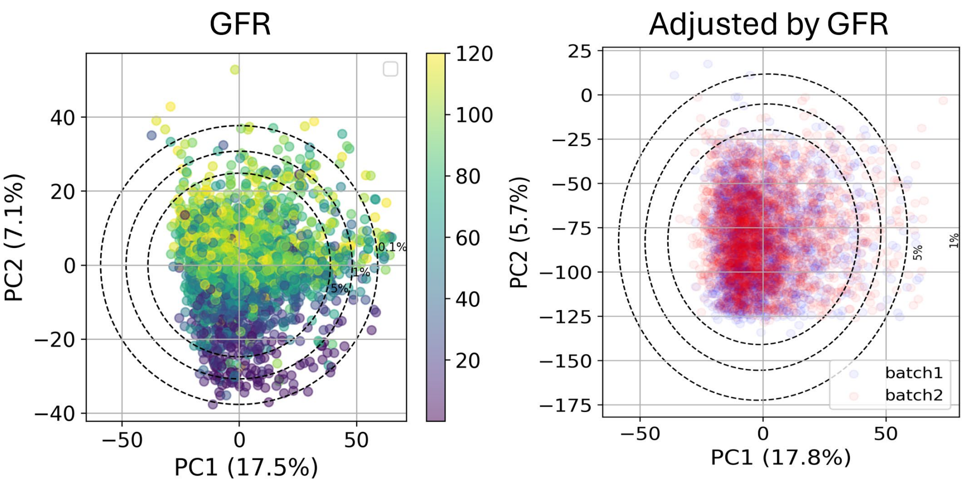

5 Harmonization of Olink and SOMAscan

5.1 Standardization of Shared Proteins

For proteins measured on both Olink and SOMAscan, we applied a $\log_2$ transformation with a small offset. Two mapping methods were used: (i) an affine transformation aligning medians and scaling by the ratio of robust standard deviations (median absolute deviations), and (ii) quantile mapping, interpolating Olink distributions to match SOMAscan quantiles.

5.2 Imputation of non-overlapping proteins

Missing proteins in UKBB were imputed using elastic-net regression trained on SOMAscan data. Overlapping proteins served as predictors and non-overlapping proteins as targets. Models were internally cross-validated, with predictors and outcomes standardized prior to fitting. The trained models were then applied to mapped Olink data to generate imputed values on the $\log_2$ scale, which were back-transformed to the linear scale. Imputed values were clipped to the 0.5th–99.5th percentile range of the SOMAscan distribution to avoid implausible extremes.

5.3 Robustness checks

Nonparametric bootstrapping with repeated resampling of the SOMAscan cohort was used to quantify uncertainty in imputation. Distributional similarity between mapped Olink and SOMAscan values was assessed using Kolmogorov–Smirnov tests, histograms, and quantile–quantile plots. The Kolmogorov–Smirnov tests showed all the P value > 0.9. Histograms and quantile–quantile plots for four representative proteins are shown below as examples.

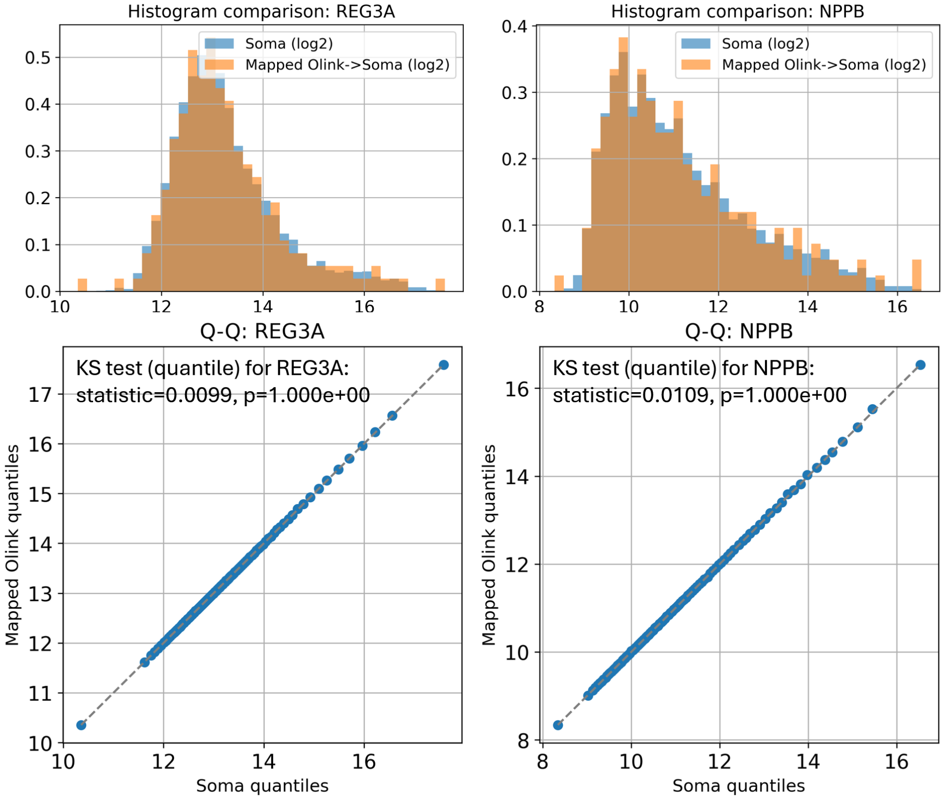

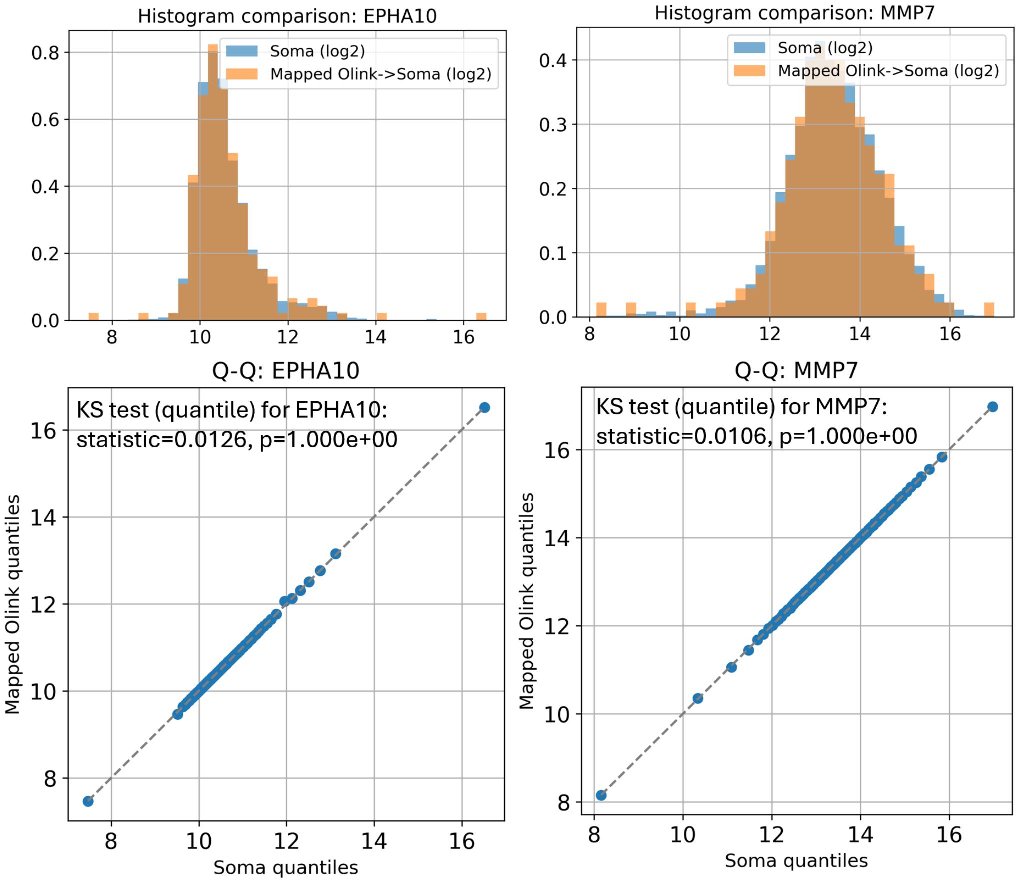

Distributional Comparison Between Mapped Olink and SOMAscan Values for four Proteins
Histograms and quantile–quantile (Q–Q) plots compare the original SOMAscan (blue) and mapped Olink-to-SOMAscan (orange) measurements. Kolmogorov–Smirnov (KS) tests demonstrated no significant distributional differences.

6 Statistical methods

6.1 Missing Data Handling

Missing data is assumed to be missing at random. Missing data was handled across the entire PMBB dataset. Variables with >15% missingness were excluded. Remaining missing values were imputed using multiple imputation by chained equations; imputation was performed separately within the training and test sets to avoid information leakage. The UACR missing rates were 5.77% for APOL1-HR participants with eGFR > 60, 6.77% for APOL1-LR participants with eGFR > 60, and 2.80% for APOL1-HR participants with eGFR ≤ 60.

6.2 Statistical Analysis for Multiplicity Correction

We will address multiple hypothesis testing by applying Benjamini-Hochberg false discovery rate (FDR) correction to all univariate analyses of proteomic biomarkers, ensuring that the overall rate of false positives remains controlled. Specifically, we will first calculate raw p-values from Cox proportional hazards models (or comparable methods) for each protein, then adjust these p-values using FDR at a predefined threshold (e.g., FDR < 0.05). In cases where a more stringent control of type I error is required—such as validating a smaller panel of

high-priority protein biomarkers—we will consider Bonferroni or Holm-Bonferroni methods. All statistical analyses will be performed using Python (including the statsmodels.stats.multitest module) and R (e.g., the p.adjust function) to maintain reproducibility. Finally, we will report effect estimates (with 95% confidence intervals) for each protein and appropriately highlight any exploratory findings that do not undergo formal multiplicity adjustment.

6.3 Cox proportional hazards model

6.3.1 Censoring and the Assumption of Independence

In survival data analysis, censoring occurs when an event is not observed due to termination of follow-up, loss to follow-up, or other reasons. To obtain unbiased estimates using Kaplan–Meier curves and Cox models, it is crucial that the censoring mechanism is independent of the actual event occurrence time. In this study, for outcomes other than death, patients censored due to loss to follow-up or the end of the 10-year follow-up period are assumed to be censored non-informatively.

Importantly, since all-cause mortality is included as an event within our composite outcome, deaths are treated as events rather than censoring. To address potential undercounting of events when using single-outcome definitions and to appropriately account for the competing risk of death, we construct a composite outcome that comprises ≥40% decline in eGFR, end-stage kidney disease, long-term dialysis, kidney transplantation, and all-cause mortality.

6.3.2 Proportional Hazards (PH) Assumption

Due to our study population being relatively healthy and the lack of significant therapeutic interventions, the potential for treatment-induced time-dependent effects is minimized. However, even in this context, factors such as aging and dynamic changes in protein expression over time could still lead to violations of the PH assumption. To validate this assumption, we will use Schoenfeld residuals tests to assess the correlation between residuals and time. Graphical diagnostics, such as log-minus-log survival plots, will also be employed to verify that the hazard functions remain parallel across groups. If any covariate exhibits evidence of non-proportionality, we plan to introduce time-dependent covariates or apply stratified Cox models to adequately adjust for such effects.

6.4 Feature Selection and modeling

6.4.1 Training and Testing Set

The datasets will be partitioned into a training set comprising 80% of APOL1-HR PMBB participants and a testing set comprising 20%. To maintain balance between the training and validation sets, we stratify based on key clinical parameters, which will be detailed in the Table of the main text.

6.4.2 Regularized Cox Regression:

We will perform a regularized Cox Regression where the L1/L2 ratio varies from 0 to 1. An alpha grid is automatically generated by sklearn and sksurv using 8-fold cross-validation, and the optimal alpha is selected based on the minimization of the partial likelihood deviance. Specifically, for a given L1 ratio, the model will be fitted using CoxnetSurvivalAnalysis (with an alpha_min_ratio set to 0.00001 and a maximum iteration of 1000), and a subset of alpha values is extracted for tuning. At each iteration we randomly sampled (with replacement) protein subsets drawn from the top 20% of proteins ranked by univariate AUC and fit the model on those subsets. This procedure was repeated across approximately 1 million different protein combinations.

6.4.3 Final modeling:

After the iterative sampling and model fitting process, the best-performing model was identified based on the lowest cross-validated partial likelihood deviance within the testing set. The final set of selected proteins and their corresponding coefficients were then used to construct the prognostic model. This finalized model was refitted on the entire training dataset to derive the final coefficients, which were subsequently applied to generate the APOL1 Proteomic Risk Score (APRS) with the clinical confounders for downstream validation and external cohort analyses.

6.5 Number needed to treat (NNT)

For a chosen horizon (e.g., 5 or 10 years), each subject will be classified as having an event by that time or not. For each score and threshold (evaluated across score percentiles), the observed event rate among subjects at or above the threshold is calculated, then multiplied by an assumed treatment relative risk reduction (RRR) to obtain the absolute risk reduction; the reciprocal of this value yields the NNT. Confidence intervals for NNT will be obtained by nonparametric bootstrap (1,000 resamples). Reported NNTs are presented across thresholds (NNT curves) and at selected operating points (e.g., the 95th percentile in the revision); thresholds that select no subjects or yield no positive risk reduction are not reported. NNTs are shown under a range of plausible RRRs (e.g., 15–60%), and a trial-informed example is also reported when an RRR is available.

```python
# pseudocode for feature selection and modeling
import numpy as np
import pandas as pd
from sklearn.pipeline import make_pipeline
from sklearn.model_selection import GridSearchCV, StratifiedKFold
from sksurv.linear_model import CoxnetSurvivalAnalysis
# Define a function to generate alpha values using CoxnetSurvivalAnalysis
def get_alpha(l1, x, y):
    c_p = make_pipeline(
        CoxnetSurvivalAnalysis(l1_ratio=l1, alpha_min_ratio=0.00001, max_iter=1000)
    )
    c_p.fit(x, y)
    return c_p.named_steps["coxnetsurvivalanalysis"].alphas_
# Define a function to build a single-layer survival analysis pipeline
def build_pipeline(l1, idx, x, y):
    X_cov = x.loc[:, idx]
    # Initialize StratifiedKFold with the event indicator for stratification
    skf = StratifiedKFold(n_splits=10, shuffle=True, random_state=0)
    # Generate candidate alpha values using the get_alpha function
    candidate_alphas = get_alpha(l1, X_cov, y)
    # Prepare parameter grid for GridSearchCV; wrap each alpha in a list
    param_grid = {"coxnetsurvivalanalysis__alphas": [[v] for v in candidate_alphas]}
    # Set up GridSearchCV with the CoxnetSurvivalAnalysis model in a pipeline
    pipeline = GridSearchCV(
        make_pipeline(CoxnetSurvivalAnalysis(l1_ratio=l1)),
        param_grid=param_grid,
        cv=skf.split(X_cov, y["event"])
    )
    return pipeline
# ------------------------- Example Data -------------------------
np.random.seed(0)
n = 50  # number of samples
x_r = pd.DataFrame({
    "feature1": np.random.randn(n),
    "PC1": np.random.randn(n),
    "N264K": np.random.randn(n),
    "sp_age": np.random.randint(30, 70, n),
    "gfr": np.random.uniform(50, 100, n)
})
event = np.random.choice([True, False], n)
time = np.random.uniform(1, 10, n)
y_struct = np.array(list(zip(event, time)), dtype=[("event", bool), ("time", float)])
# Define idx as a list with one feature for the model
idx = ["feature1"]
l1 = 0.5
pipeline = build_pipeline(l1, idx, x_r, y_struct)
pipeline.fit(x_r.loc[:, idx].fillna(0), y_struct)
print("Best parameters:", pipeline.best_params_)
best_model = pipeline.best_estimator_.named_steps["coxnetsurvivalanalysis"]
print("Coefficients:", best_model.coef_)
```

7 Model Performance Evaluation

We will assess the predictive model using survival analysis metrics. The evaluation function takes as input the true survival outcomes (for both training and test sets), predicted risk scores, and the prediction time points. The process is as follows:

1. **Data Preparation**

   Align predicted risk scores with true survival outcomes.

   Apply inverse probability of censoring weights (IPCW) on the test set to adjust for censoring. The IPCW for sample *i* at time *t* is calculated as: $IPCW_{i,t} = \frac{1}{\hat{s}(t|X_i)}$ where $\hat{S}(t|X_i)$ is the estimated probability of being uncensored at time *t* given the covariates X_i.

2. **Metric Calculation at Each Time Point**

   Sort samples in descending order of risk scores.

   For each time point *t*, define:

   - **TP** (true positives)
   - **FP** (false positives)
   - **TN** (true negatives)
   - **FN** (false negatives)

   In the computation of these quantities, the IPCW is applied to the counts of events. For instance, the true positive count is computed as the sum of IPCW-adjusted indicators for cases (i.e., if an event occurs before or at time *t*).

   Compute performance metrics including:

   - **AUC**: $AUC_t = \int_0^1 TPR_t(FPR_t)\,, dFPR_t$where TPR_t is the true positive rate (sensitivity) and FPR_t is the false positive rate (1 - specificity) at time t
   - **Sensitivity**: $Sensitivity_t = \frac{TP_t}{TP_t+FN_t}$
   - **Specificity**: $Specificity_t = \frac{TN_t}{TN_t+FP_t}$,
   - **F1 Score**: $F1_t = \frac{2 \cdot Precision_t \cdot Recall_t}{Precision_t+Recall_t}$
   - **Precision:** $Precision_t = \frac{TP_t}{TP_t+FP_t}$
   - **Accuracy**: $Accuracy_t = \frac{TP_t+TN_t}{TP_t+FP_t+TN_t+FN_t}$
   - **Recall:** $Recall_t = \frac{TP_t}{TP_t+FN_t}$
   - **Matthews Correlation Coefficient (MCC)**:

$$MCC_t = \frac{\big((TP_t * TN_t) - (FP_t * FN_t)\big)}{\sqrt{(TP_t + FP_t) * (TP_t + FN_t) * (TN_t + FP_t) * (TN_t + FN_t)}}$$

- **Positive Predictive Value (PPV)** and **Negative Predictive Value (NPV)**:

  $PPV_t = \frac{TP_t}{TP_t+FP_t}$ and $NPV_t = \frac{TN_t}{TN_t+FN_t}$

The binary classification threshold at each time point is chosen to maximize the sum of sensitivity and specificity.

3. **Weighted Aggregation (for Multiple Time Points)**

If multiple time points are evaluated, compute the weighted mean of each metric using the survival probability at each time point

$$\bar{M} = \frac{\sum_{t=1}^{m} M_t \cdot \hat{S}(t)}{\sum_{t=1}^{m} \hat{S}(t)}$$

where M_t is the metric at time *t* and $\hat{S}(t)$ is the estimated survival probability.

The function returns a dictionary containing the performance metrics at each time point, along with the overall weighted mean metrics if applicable. The pseudo code is as follows:

```python
import numpy as np
from sksurv.util import check_y_survival
from sksurv.nonparametric import CensoringDistributionEstimator, SurvivalFunctionEstimator
def cum_metrics(survival_train, survival_test, estimate, times, AUC_sfd=False,
                tied_tol=1e-8):
    test_event, test_time = check_y_survival(survival_test)
    event_shuffle = test_event
    estimate, times = _check_estimate_2d(estimate, test_time, times,
                                         estimator="cumulative_dynamic_auc")
    n_samples = estimate.shape[0]
    n_times = times.shape[0]
    if estimate.ndim == 1:
        estimate = np.broadcast_to(estimate[:, np.newaxis], (n_samples, n_times))
    cens = CensoringDistributionEstimator()
    cens.fit(survival_train)
    ipcw = cens.predict_ipcw(survival_test)
    test_time = np.broadcast_to(test_time[:, np.newaxis], (n_samples, n_times))
    test_event = np.broadcast_to(test_event[:, np.newaxis], (n_samples, n_times))
    times_2d = np.broadcast_to(times, (n_samples, n_times))
    ipcw = np.broadcast_to(ipcw[:, np.newaxis], (n_samples, n_times))
    o = np.argsort(-estimate, axis=0)
    test_time = np.take_along_axis(test_time, o, axis=0)
    test_event = np.take_along_axis(test_event, o, axis=0)
    estimate = np.take_along_axis(estimate, o, axis=0)
    ipcw = np.take_along_axis(ipcw, o, axis=0)
    is_case = (test_time <= times_2d) & test_event
    is_control = test_time > times_2d
    n_controls = is_control.sum(axis=0)
    estimate_diff = np.concatenate((np.broadcast_to(np.infty, (1, n_times)),
                                    estimate))
    is_tied = np.absolute(np.diff(estimate_diff, axis=0)) <= tied_tol
    CU_tp = np.cumsum(is_case * ipcw, axis=0)
    CU_fp = np.cumsum(is_control, axis=0)
    CU_tn = n_controls - CU_fp
    CU_fn = CU_tp[-1] - CU_tp
    CU_n_neg = CU_tn + CU_fn
    CU_n_pos = CU_tp + CU_fp
    tpr_all = CU_tp / CU_tp[-1]  # sensitivity
    fpr_all = CU_fp / (CU_tn + CU_fp)  # 1 - specificity
    pr_all = CU_tp / (CU_tp + CU_fp + 1)
    tnr_all = 1 - pr_all
    ac_all = (CU_tp + CU_tn) / (CU_tn + CU_fn + CU_tp + CU_fp)
    fnr_all = CU_fn / (CU_tp + CU_fn)
    mmc_all = ((CU_tn * CU_tp) - (CU_fp * CU_fn)) / \
        np.sqrt((CU_tn + CU_fn) * (CU_fp + CU_tp) * (CU_tn + CU_fp) * (CU_fn + CU_tp))
    prev_all = (CU_tp + CU_fn) / (CU_tn + CU_fn + CU_tp + CU_fp)
    ppv_all = ((tpr_all) * prev_all) / ((tpr_all * prev_all) + ((fpr_all) * (1 - prev_all)))
    npv_all = ((1 - fpr_all) * (1 - prev_all)) / (((1 - tpr_all) * prev_all) + \
                                                  ((1 - fpr_all) * (1 - prev_all)))
    res_keys = ["AUC", "AUC_P", "best_spe", "best_sen", "f1_scores", "best_pr",
```

```python
                "best_rec", "best_ac","best_fpr", "best_fnr", "best_mcc", "best_ppv",
                "best_npv"]
    res = {key: np.empty(n_times, dtype=float) for key in res_keys}
    it = np.nditer((tpr_all, fpr_all, pr_all, ac_all, fpr_all, fnr_all,
                    mmc_all, ppv_all, npv_all, CU_n_pos, CU_n_neg, is_tied), order="F",
                    flags=["external_loop"])
    with it:
        for i, (tpr, fpr, pr, ac, fpr2, fnr, mmc, ppv, npv, n_pos, n_neg, mask) in enumerate(it):
            idx = np.flatnonzero(mask) - 1
            tpr_no_ties = np.r_[0, np.delete(tpr, idx)]
            fpr_no_ties = np.r_[0, np.delete(fpr, idx)]
            pr_no_ties = np.r_[0, np.delete(pr, idx)]
            res['AUC'][i] = np.trapz(tpr_no_ties, fpr_no_ties)
            if type(AUC_sfd) != bool:
                res['AUC_P'][i] = (np.sum(AUC_sfd[:, i] >= res['AUC'][i]) + 1) / \
                    (len(AUC_sfd[:, i]) + 1)
            best_index = np.argmax(tpr_no_ties + (1 - fpr_no_ties))
            res['best_sen'][i] = tpr_no_ties[best_index]
            res['best_spe'][i] = 1 - fpr_no_ties[best_index]
            res['best_ac'][i] = np.r_[0, np.delete(ac, idx)][best_index]
            res['best_fpr'][i] = fpr_no_ties[best_index]
            res['best_fnr'][i] = np.r_[0, np.delete(fnr, idx)][best_index]
            res['best_mcc'][i] = np.r_[0, np.delete(mmc, idx)][best_index]
            res['best_ppv'][i] = np.r_[0, np.delete(ppv, idx)][best_index]
            res['best_npv'][i] = np.r_[0, np.delete(npv, idx)][best_index]
            f1 = 2 * (pr_no_ties * tpr_no_ties) / (pr_no_ties + tpr_no_ties + \
                                                  np.finfo(float).eps)
            res['best_pr'][i] = pr_no_ties[np.argmax(f1)]
            res['best_rec'][i] = tpr_no_ties[np.argmax(f1)]
            res['f1_scores'][i] = f1[np.argmax(f1)]
    if n_times == 1:
        return res, []
    else:
        surv = SurvivalFunctionEstimator()
        surv.fit(survival_test)
        s_times = surv.predict_proba(times)
        d = -np.diff(np.r_[1.0, s_times])
        res_mean = {key: (res[key] * d).sum() / (1.0 - s_times[-1]) for key in res_keys}
    return res, res_mean

# ======================= Test Data Generation =======================
if __name__ == '__main__':
    np.random.seed(42)
    n_train = 30
    n_test = 20
    train_events = np.random.choice([True, False], size=n_train, p=[0.7, 0.3])
    train_times = np.random.uniform(1, 20, size=n_train)
    survival_train = np.array(list(zip(train_events, train_times)),
                              dtype=[('event', 'bool'), ('time', 'float')])
    test_events = np.random.choice([True, False], size=n_test, p=[0.5, 0.5])
```

```
    test_times = np.random.uniform(1, 20, size=n_test)
    survival_test = np.array(list(zip(test_events, test_times)),
                             dtype=[('event', 'bool'), ('time', 'float')])
    n_times = 5
    estimate = np.random.rand(n_test, n_times)
    times = np.linspace(1, 20, n_times)
    res, res_mean = cum_metrics(survival_train, survival_test, estimate, times,
AUC_sfd=False)
    print("Detailed metrics for each time point:")
    for key, value in res.items():
        print(f"{key}: {value}")

    if res_mean:
        print("\nMean metrics (integrated over time):")
        for key, value in res_mean.items():
            print(f"{key}: {value}")
```

8 Sample Size and Power

For sample size calculation, we employed two approaches. The first approach involves screening the proteins, while the second focuses on constructing a predictive model. Both approaches are based on the Cox proportional hazards (Cox PH) model.

8.1 Sample size for screening the proteins

Sample size estimation for screening the proteins base the Cox proportional hazards model was performed using the Schoenfeld formula. The required number of events D is given by:

$$D = \frac{\left(z_{1-\alpha/2} + z_{1-\beta}\right)^2}{p(1-p)[\ln(\mathrm{HR})]^2},$$

where:

- α is the two-sided significance level (set to 0.05, so that $z_{1-\alpha/2} \approx 1.96$).
- β is the Type II error rate (with statistical power $= 1 - \beta$).
- p is the expected proportion of subjects who with marker-positive (in this study, 30% or $p = 0.3$).
- $\ln(\mathrm{HR})$ is the natural logarithm of the hazard ratio to be detected.

The total sample size N is then calculated by adjusting the required number of events by the expected event rate during follow-up: $N = \frac{D}{\text{event rate}}$

In our study, we assumed:

- an expected event rate of 25% (i.e., 0.25),
- an anticipated hazard ratio of approximately 1.648 (which is close to exp(0.5)).

Under these assumptions, enrolling 685 subjects would yield an effective statistical power of about 85% for detecting a hazard ratio of 1.648 (Figure 1). The pseudo code is as follows:

```python
import math
import numpy as np
import matplotlib.pyplot as plt
from scipy.stats import norm
import pandas as pd
def calculate_sample_size_cox(alpha, power, hr, p=0.5, event_rate=1.0):
    """
    Calculate the required sample size for a Cox proportional hazards model
    based on the Schoenfeld formula.
    Parameters:
        alpha (float): Significance level (Type I error rate), e.g., 0.05.
        power (float): Statistical power (1 - beta), e.g., 0.8.
        hr (float): Hazard ratio to detect, e.g., 1.65.
        p (float): Proportion of subjects in the designated group (default is 0.5).
        event_rate (float): Expected event rate during the study period (default is 1.0, meaning all subjects experience the event).
    Returns:
        n (int): Estimated total sample size required.
    """
    # Calculate the z-scores for the given alpha and power
    z_alpha = norm.ppf(1 - alpha / 2)
    z_power = norm.ppf(power)
    # Compute the required number of events using Schoenfeld's formula
    d = ((z_alpha + z_power) ** 2) / (p * (1 - p) * (math.log(hr)) ** 2)
    # Total sample size adjusted by the expected event rate
    n = d / event_rate
    # Return the sample size rounded up to the next integer
    return math.ceil(n)
# Use ggplot style for the plot
plt.style.use('ggplot')
# Define fixed parameters
alpha = 0.05          # Significance level
p = 0.3               # Proportion in the designated group
event_rate = 0.25     # Expected event rate during follow-up
# Create an array of hazard ratios ranging from 1.4 to 2.0
hr_values = np.linspace(1.4, 2.0, 100)
# Define multiple power values for comparison
power_values = [0.8, 0.85, 0.9]
# Plotting the relationship between hazard ratio and required sample size for different power values
plt.figure(figsize=(6, 6))
# Draw a horizontal line at sample size = 700
plt.axhline(y=700, color='red', linestyle='--', linewidth=2, label='700 People')
# Draw a vertical line at HR = exp(0.5) ≈ 1.65
plt.axvline(x=1.65, color='blue', linestyle='--', linewidth=2, label='exp(0.5)')
res=[]
for power in power_values:
    sample_sizes = [calculate_sample_size_cox(alpha, power, hr, p, event_rate) for hr in hr_values]
    plt.plot(hr_values, sample_sizes, linewidth=2, label=f'Power = {power}')
    res_s=pd.DataFrame({'HR': hr_values, 'Sample Size': sample_sizes})
    res_s['Power']=power
    res.append(res_s)
plt.xlabel('Hazard Ratio (HR)')
plt.ylabel('Required Total Sample Size')
plt.title('Sample Size vs Hazard Ratio for Cox PH Model')
plt.legend(title='Statistical Power')
plt.grid(True)
plt.show()
res=pd.concat(res)
res.to_csv('sample_size_vs_hr.csv', index=False)
```

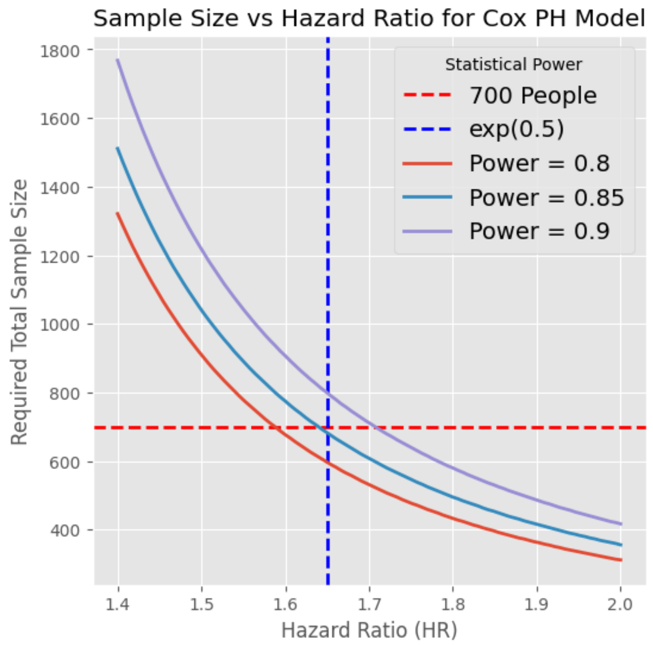

Sample Size vs. Hazard Ratio for Cox Proportional Hazards Model. The plot displays curves of the required total sample size for detecting a given hazard ratio (HR) under different statistical power settings (80%, 85%, and 90%) using the Schoenfeld formula. The horizontal red dashed line represents a total sample size of 700 subjects, while the vertical blue dashed line marks a hazard ratio of approximately 1.65 (equivalent to exp(0.5)).

8.2 Sample Size Estimation for Predictive Model Construction

In developing our predictive model based on the Cox proportional hazards framework, we employed two complementary approaches for sample size determination: Riley's method and the traditional EPV (events per variable) rule. These methods were used to ensure the robustness of the model while minimizing the risk of overfitting.

Riley's Method

Riley's method[8] is an advanced approach that calculates the required sample size by considering model performance parameters and anticipated shrinkage. Using the R package *pmsampsize*, we computed the required total sample size for a range of candidate predictor numbers (denoted by k). The calculations incorporated the following parameters:

- A candidate model's anticipated performance was characterized by a global R^2, which was derived using the formula:

$$R_{CS}^2 = \frac{\left(R_{app} - 0.5\right)^2}{0.75}$$

where R_{app} represents the apparent R^2 (in our case, 0.88).

- An expected event rate of 25% was assumed.

- A time horizon of 10 years for the outcome and a mean follow-up of 10 years were specified.

For each value of k, Riley's method provided an estimate of the required total sample size to achieve a pre-specified level of predictive performance while controlling for overfitting. These estimates were then compared across values of k.

EPV-Based Sample Size Calculation

In parallel, we applied the widely used rule-of-thumb based on the events per variable (EPV) criterion. According to this approach, a minimum number of events per predictor is required to reliably estimate model parameters. The required number of events D is given by:

$$D = EPV \times k$$

where EPV is typically set to 10 (or adjusted to 11 or 12 in sensitivity analyses) and k represents the number of candidate predictors. Given an expected event rate (denoted as λ), the total required sample size N is then calculated as:

$$N = \frac{D}{\lambda} = \frac{(EPV \times k)}{\lambda}$$

For example, assuming an event rate of 25%, the total sample size for an EPV of 10 would be computed as:

$$N = \frac{10 \times k}{0.25}$$

This calculation was performed across a range of k values, and similar computations were repeated for EPV values of 11 and 12 to assess the sensitivity of the sample size requirement (Figure 2).

In our study, we assumed:

- an expected event rate of 25% (i.e., 0.25),
- candidate predictor numbers ranging up to 15, and
- performance parameters as specified by the global R^2, whereR_{app}equal to 0.88

The required total sample sizes were:

- EPV = 10: sample size = 640,
- EPV = 11: sample size = 704,
- EPV = 12: sample size = 768.
- Riley's method sample size = 782

Based on these estimates, we ultimately adopted a sample size of 700 participants. This choice is consistent with our initial sample size estimation for protein screening and is expected to provide sufficient power for robust predictive modeling while minimizing the risk of overfitting. The pseudo code is as follows:

```
import math
import numpy as np
import pandas as pd
import matplotlib.pyplot as plt
################################################################################
# Create a DataFrame with your Riley results
'''## R Riley's Method
library(pmsampsize)
parameters <- 1:20
sample_sizes <- vector("list", length(parameters))
# Loop over each parameter value
for (i in parameters) {
  # Call pmsampsize with the current parameter and store the result in res
  res <- pmsampsize(type = "s", csrsquared = (0.88 - 0.5)^2 / 0.75, parameters = i,
    rate = 0.24,timepoint = 10, meanfup = 10)
  # Use double bracket [[3]] to extract the third element as a whole vector
  sample_sizes[[i]] <- res[[3]]
}
result_Riley <- data.frame(Parameter = parameters, SampleSize = I(sample_sizes))
'''
df_riley = pd.DataFrame(riley_data)
k_values_riley = df_riley["Parameter"].values
sample_sizes_riley = df_riley["SampleSize"].values
################################################################################
def calculate_sample_size_epv(k, EPV, event_rate):
    events_required = EPV * k
    # Total sample size = required events / event_rate
    sample_size = events_required / event_rate
    return math.ceil(sample_size)
# Set the event rate and compute EPV-based sample sizes for different EPV values
event_rate = 0.25  # Example: 25% event rate
sample_sizes_epv_10 = [calculate_sample_size_epv(k, 10, event_rate) for k in k_values_riley]
sample_sizes_epv_11 = [calculate_sample_size_epv(k, 11, event_rate) for k in k_values_riley]
sample_sizes_epv_12 = [calculate_sample_size_epv(k, 12, event_rate) for k in k_values_riley]
# Plot everything: multiple EPV lines (no markers) plus the Riley line
plt.style.use("ggplot")
plt.figure(figsize=(6, 6))
plt.plot(k_values_riley,sample_sizes_riley,linewidth=2,marker=None, label="Riley's Method")
plt.plot(k_values_riley,sample_sizes_epv_10,linewidth=2,marker=None, label="EPV=10")
plt.plot(k_values_riley,sample_sizes_epv_11,linewidth=2,marker=None, label="EPV=11")
plt.plot(k_values_riley,sample_sizes_epv_12,linewidth=2,marker=None, label="EPV=12")
plt.axhline(y=700, color='red', linestyle='--', linewidth=2, label='700 People')
plt.axvline(x=15, color='blue', linestyle='--', linewidth=2, label='15 markers')
plt.xlabel("Number of Candidate Predictors (k)")
plt.ylabel("Required Total Sample Size")
plt.title("Sample Size Estimation: Riley's Method vs. EPV Rule")
plt.legend(title="Method")
plt.grid(True)
plt.show()
```

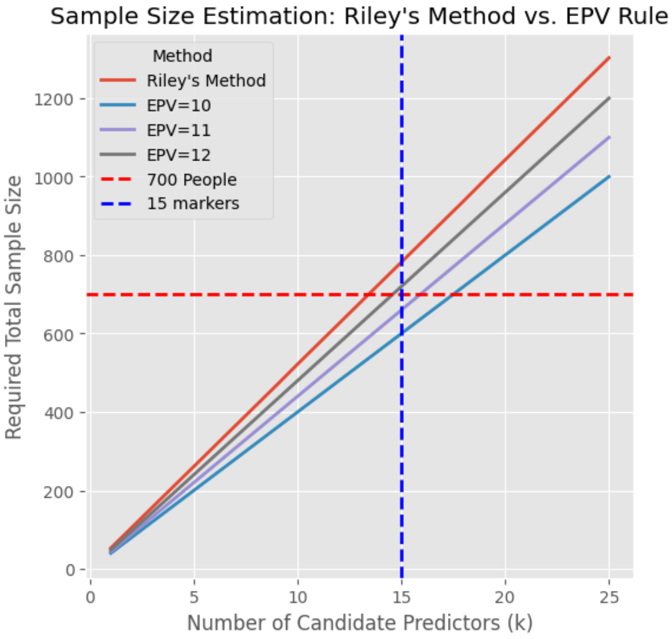

Sample Size Estimation Using Riley's Method and the EPV Rule.
The figure illustrates the required total sample size for predictive modeling as a function of the number of candidate predictors (k), estimated using both Riley's method (solid red line) and the EPV rule with different assumptions (EPV = 10, 11, and 12, represented by solid black, blue, and green lines, respectively). Riley's method accounts for model performance characteristics and expected shrinkage, while the EPV rule provides a heuristic estimation based on the number of events per variable. The dashed red horizontal line indicates a reference sample size of 700 participants, and the dashed blue vertical line corresponds to a scenario with 17 candidate predictors. These reference lines aid in visualizing feasible study designs under different methodological assumptions.

Reference

1. Genovese G, Friedman DJ, Ross MD, et al. Association of trypanolytic ApoL1 variants with kidney disease in African Americans. Science 2010;329:841-5.
2. Parsa A, Kao WH, Xie D, et al. APOL1 risk variants, race, and progression of chronic kidney disease. N Engl J Med 2013;369:2183-96.
3. Tangri N, Grams ME, Levey AS, et al. Multinational Assessment of Accuracy of Equations for Predicting Risk of Kidney Failure: A Meta-analysis. JAMA 2016;315:164-74.
4. Egbuna O, Zimmerman B, Manos G, et al. Inaxaplin for Proteinuric Kidney Disease in Persons with Two APOL1 Variants. N Engl J Med 2023;388:969-79.
5. Kammer M, Heinzel A, Willency JA, et al. Integrative analysis of prognostic biomarkers derived from multiomics panels helps discrimination of chronic kidney disease trajectories in people with type 2 diabetes. Kidney Int 2019;96:1381-8.
6. Gaster T, Eggertsen CM, Stovring H, Ehrenstein V, Petersen I. Quantifying the impact of unmeasured confounding in observational studies with the E value. BMJ Med 2023;2:e000366.
7. Langfelder P, Horvath S. WGCNA: an R package for weighted correlation network analysis. BMC Bioinformatics 2008;9:559.
8. Riley RD, Ensor J, Snell KIE, et al. Calculating the sample size required for developing a clinical prediction model. BMJ 2020;368:m441.
