# Supplementary material for: Proteomic risk score for early prediction of kidney disease progression in individuals with APOL1 high-risk genotypes
Source: Nat Med. 2026 Apr 15;32(5):1701–7. doi: 10.1038/s41591-026-04337-2 (PMC13190321; doi:10.1038/s41591-026-04337-2)
Supplement: Supplementary file 2 — Reporting Summary [file 41591_2026_4337_MOESM2_ESM.pdf]

nature portfolio

Corresponding author(s): Katalin Susztak
Last updated by author(s): Feb 25, 2026

Reporting Summary

Nature Portfolio wishes to improve the reproducibility of the work that we publish. This form provides structure for consistency and transparency in reporting. For further information on Nature Portfolio policies, see our Editorial Policies and the Editorial Policy Checklist.

Statistics

For all statistical analyses, confirm that the following items are present in the figure legend, table legend, main text, or Methods section.

n/a	Confirmed	
☐	☒	The exact sample size (*n*) for each experimental group/condition, given as a discrete number and unit of measurement
☐	☒	A statement on whether measurements were taken from distinct samples or whether the same sample was measured repeatedly
☐	☒	The statistical test(s) used AND whether they are one- or two-sided<br>*Only common tests should be described solely by name; describe more complex techniques in the Methods section.*
☐	☒	A description of all covariates tested
☐	☒	A description of any assumptions or corrections, such as tests of normality and adjustment for multiple comparisons
☐	☒	A full description of the statistical parameters including central tendency (e.g. means) or other basic estimates (e.g. regression coefficient) AND variation (e.g. standard deviation) or associated estimates of uncertainty (e.g. confidence intervals)
☐	☒	For null hypothesis testing, the test statistic (e.g. *F*, *t*, *r*) with confidence intervals, effect sizes, degrees of freedom and *P* value noted<br>*Give P values as exact values whenever suitable.*
☒	☐	For Bayesian analysis, information on the choice of priors and Markov chain Monte Carlo settings
☐	☒	For hierarchical and complex designs, identification of the appropriate level for tests and full reporting of outcomes
☐	☒	Estimates of effect sizes (e.g. Cohen's *d*, Pearson's *r*), indicating how they were calculated

Our web collection on statistics for biologists contains articles on many of the points above.

Software and code

Policy information about availability of computer code

Data collection	The PMBB WES Release 3.0 used bcl2fastq (Illumina), BWA-MEM, DeepVariant v0.10 (Parabricks-accelerated), GLnexus v1.4.3, PLINK v1.9, bcftools norm, VEP v113.0 with plugins (AlphaMissense, CADD, ClinVar, dbNSFP 4.9, gnomAD, LOFTEE, SpliceAI), KING, R (KernSmooth), Python, smartpca (EIGENSOFT), and the SAIGE-GENE pipeline (Nextflow). STAR (v2.7.3a) was used to align the bulk RNA-seq data to the human genome.Proteomic profiling was performed using SOMAscan v4.1 with data processed in the SOMAscan Data Analysis Software (SomaLogic) and microarrays scanned on the SureScan Dx Microarray Scanner (Agilent Technologies).
Data analysis	For RNA-seq data processing, TrimGalore (v0.4.5) was used for trimming adaptors and low-quality bases, and STAR (v2.7.3a) was employed for aligning reads to the human genome. RSEM (v1.3.0) was used to estimate gene and isoform expression levels in transcripts per million. The Python environment for this analysis included lifelines 0.28.0 for survival analysis, matplotlib 3.7.5 for plotting, numpy 1.24.4 and scipy 1.9.3 for numerical and statistical computations, pandas 1.5.3 for data manipulation, scikit-learn 1.3.2 for model building, sksurv 0.22.2 for survival models with censored data, and all running on Python 3.10.12 (GCC 11.4.0).

For manuscripts utilizing custom algorithms or software that are central to the research but not yet described in published literature, software must be made available to editors and reviewers. We strongly encourage code deposition in a community repository (e.g. GitHub). See the Nature Portfolio guidelines for submitting code & software for further information.

Data

Policy information about availability of data

All manuscripts must include a data availability statement. This statement should provide the following information, where applicable:

- Accession codes, unique identifiers, or web links for publicly available datasets
- A description of any restrictions on data availability
- For clinical datasets or third party data, please ensure that the statement adheres to our policy

The datasets analyzed in this study are not publicly available due to participant privacy and data use agreements but may be accessed upon reasonable application to the PMBB (https://pmbb.med.upenn.edu/), subject to approval. Requests for proteomic data or verification analyses may be directed to the corresponding author (ksusztak@pennmedicine.upenn.edu). An initial response is generally provided within approximately two weeks. ARIC data may be requested from the ARIC Data Coordinating Center by obtaining study approval, executing a Data and Materials Distribution Agreement, and submitting a Data Request Form to aricdata@unc.edu. Alternatively, ARIC data are available through the NHLBI BioLINCC (https://biolincc.nhlbi.nih.gov/) repository and dbGaP subject to their application procedures (https://dbgap.ncbi.nlm.nih.gov/beta/study/phs000280.v9.p3). Review timelines typically require approximately 4–8 weeks. For datasets derived from the UKBB, data access is governed by the UK Biobank's established policies. Researchers must apply through the UKBB Access Management System, available at https://www.ukbiobank.ac.uk/, outlining the purpose of the intended use. Applications are reviewed by UK Biobank, and decisions are generally provided within approximately 4–6 weeks.

Research involving human participants, their data, or biological material

Policy information about studies with human participants or human data. See also policy information about sex, gender (identity/presentation), and sexual orientation and race, ethnicity and racism.

Reporting on sex and gender	For PMBB cohort, sex was determined based on genetic data obtained from whole genome sequencing for the study participants. The data included information on participants being male or female; there were no sex/gender-based inclusion or exclusion criteria.
Reporting on race, ethnicity, or other socially relevant groupings	Race and ethnicity information was determined based on genetic data obtained from whole genome sequencing for the study participants.
Population characteristics	Baseline characteristics are described in Table 1, Supplemental Table 1 and Extended Data Table 4.
Recruitment	The PMBB, which has enrolled over 250,000 participants since 2008, has performed whole exome sequencing on approximately 57,170 individuals. Of these, 1,310 carried the high-risk APOL1 genotype, and after excluding 197 participants with prior kidney transplantation or kidney failure, 1,113 African American participants (G1/G1, G2/G2, or G1/G2) were included in this study. Additional 912 African ancestry APOL1 low-risk participants and 698 European ancestry APOL1 low-risk were included as external reference.
Ethics oversight	The data and biospecimen used in this study in PMBB were approved by the University of Pennsylvania Institutional Review Board (protocol 815796, 813913 and 857403) and all participants provided informed consent for genetic and EHR research. The validation approved by the University of Pennsylvania Institutional Review Board (protocol 855821). ARIC and UK Biobank investigations were conducted under their respective ethics approvals (UKB ethics: https://www.ukbiobank.ac.uk/about-us/how-we-work/ethics/ ; ARIC application MP4524; UKB application 273810).<br><br>The ARIC Study adhered to ethics regulations from and was approved by a single Institutional Review Board at Johns Hopkins School of Medicine (FWA00005752; IRB00311861) and Institutional Review Boards at all participating institutions: University of North Carolina at Chapel Hill, Johns Hopkins University School of Public Health, University of Minnesota, Wake Forest University Health Sciences, University of Mississippi Medical Center, Baylor College of Medicine, University of Texas Houston Health Science Center, and Brigham and Women's Hospital. Study participants provided written informed consent at all study visits.<br><br>All procedures will comply with the Declaration of Helsinki and applicable regulations. Participant privacy will be protected by using de-identified data, secure servers with encryption, and HIPAA-compliant protocols. Data use agreements restrict access to authorized personnel, and data transfer (for validation analyses) will employ secure, encrypted channels.

Note that full information on the approval of the study protocol must also be provided in the manuscript.

Field-specific reporting

Please select the one below that is the best fit for your research. If you are not sure, read the appropriate sections before making your selection.

- [x] Life sciences
- [ ] Behavioural & social sciences
- [ ] Ecological, evolutionary & environmental sciences

For a reference copy of the document with all sections, see nature.com/documents/nr-reporting-summary-flat.pdf

Life sciences study design

All studies must disclose on these points even when the disclosure is negative.

Sample size	Sample size was estimated using the Schoenfeld method for the Cox proportional hazards model and an events per variable (EPV) approach (with EPV set at 15). The EPV method yielded the required sample size of 640 participants. In contrast, based on a two-sided significance level of 0.05, an expected 30% marker-positive rate, an anticipated event rate of 25%, and a target hazard ratio of 1.648—assuming median dichotomization of the model score, a total of 685 participants was determined to provide an effective statistical power of ~85%.
Data exclusions	We excluded 197 participants with prior kidney transplantation or kidney failure.
Replication	The APRS was independently validated in the Atherosclerosis Risk in Communities study and the UK Biobank, and the analyses in each cohort were performed independently by separate analysts.<br>To ensure reproducibility and consistent results, all analyses were conducted using fixed random seeds (42 or 0), implemented in Python using random.seed() and numpy.random.seed().
Randomization	This is not application as this was an observational cohort study.
Blinding	Blinding was not applicable to this study.

Reporting for specific materials, systems and methods

We require information from authors about some types of materials, experimental systems and methods used in many studies. Here, indicate whether each material, system or method listed is relevant to your study. If you are not sure if a list item applies to your research, read the appropriate section before selecting a response.

Materials & experimental systems

n/a	Involved in the study
☒	☐ Antibodies
☒	☐ Eukaryotic cell lines
☒	☐ Palaeontology and archaeology
☒	☐ Animals and other organisms
☒	☐ Clinical data
☒	☐ Dual use research of concern
☒	☐ Plants

Methods

n/a	Involved in the study
☒	☐ ChIP-seq
☒	☐ Flow cytometry
☒	☐ MRI-based neuroimaging

Plants

Seed stocks	*Report on the source of all seed stocks or other plant material used. If applicable, state the seed stock centre and catalogue number. If plant specimens were collected from the field, describe the collection location, date and sampling procedures.*
Novel plant genotypes	*Describe the methods by which all novel plant genotypes were produced. This includes those generated by transgenic approaches, gene editing, chemical/radiation-based mutagenesis and hybridization. For transgenic lines, describe the transformation method, the number of independent lines analyzed and the generation upon which experiments were performed. For gene-edited lines, describe the editor used, the endogenous sequence targeted for editing, the targeting guide RNA sequence (if applicable) and how the editor was applied.*
Authentication	*Describe any authentication procedures for each seed stock used or novel genotype generated. Describe any experiments used to assess the effect of a mutation and, where applicable, how potential secondary effects (e.g. second site T-DNA insertions, mosiacism, off-target gene editing) were examined.*
